# Mental and physical health and well-being of canadian employees who were working from home during the COVID-19 pandemic

**DOI:** 10.1186/s12889-022-14349-5

**Published:** 2022-10-31

**Authors:** Kumara G. Somasundram, Amy Hackney, Marcus Yung, Bronson Du, Jodi Oakman, Behdin Nowrouzi-Kia, Amin Yazdani

**Affiliations:** 1 Canadian Institute for Safety, Wellness & Performance, School of Business, Conestoga College Institute of Technology and Advanced Learning, Kitchener, ON Canada; 2 Centre for Ergonomics and Human Factors, School of Psychology & Public Health, La Trobe University, Bundoora, Australia; 3 Department of Occupational Science and Occupational Therapy, University of Toronto, Toronto, ON Canada; 4 School of Public Health and Health Systems, University of Waterloo, Waterloo, ON Canada; 5 School of Geographic and Earth Sciences, McMaster University, Hamilton, ON Canada

**Keywords:** Remote work, Telework, COPSOQ, National surveys, Within-individual analysis

## Abstract

**Background:**

The COVID-19 pandemic has drastically changed various aspects of our lives, including how we work. Since the start of the pandemic, numerous organizations in Canada have mandated their employees to work from home (WFH) on a full-time basis. The rapid rise in the number of remote workers and the possibility for WFH continuing in the future signifies the importance of understanding the health and well-being of employees working from home over the course of the pandemic in Canada. We present the findings of two surveys (initial and 6-month follow-up) to examine the health and well-being of WFH employees during the COVID-19 pandemic in Canada. We analyzed the changes in mental and physical health and well-being of employees who were working from home between two time points during the pandemic.

**Methods:**

Initial survey was completed between October 2020 and December 2020 (n = 1617); follow-up survey was completed between May 2021 and June 2021 (n = 382). We calculated the frequencies for survey questions involving demographics, WFH preferences, workstation setup training, employment situation, provision of hardware technologies, provision and usage of software technologies, and organization’s return to work plan. We conducted Wilcoxon signed-rank tests to analyze the within-individual changes in mental and physical health and well-being of the 382 respondents who completed both the initial and follow-up surveys.

**Results:**

Our analyses showed significant changes in various aspects of employee mental and physical health and well-being. Burnout, stress, general mental health, and job insecurity levels significantly decreased between the two time periods. Work-related sedentary behaviour reduced over time; however, the average proportion of time spent sitting during work hours was more than 80% in both surveys. Employees received more help and feedback from their colleagues and experienced a better sense of community with their co-workers over time.

**Conclusion:**

The findings can inform workers and organizations on the changes in mental and physical health and well-being of employees working from home during the pandemic. By understanding the changes in worker health and well-being, employers can develop effective strategies and implement policies that help protect employees’ health and well-being.

**Supplementary Information:**

The online version contains supplementary material available at 10.1186/s12889-022-14349-5.

## Introduction

The COVID-19 pandemic has drastically affected the lives of Canadians. On March 11, 2020, the World Health Organization (WHO) declared COVID-19 as a global pandemic, and as of May 2022, there had been over 3.7 million reported cases since the start of the pandemic in Canada. To minimize the spread of the virus, the government announced a series of lockdowns and as a result, many organizations have mandated their employees to work from home (WFH). Prior to the COVID-19 pandemic, only 4% of the Canadian workforce worked remotely; however, the proportion increased to nearly 31% during the pandemic [1]. Similar trends were observed worldwide [2–4].

It is likely that working from home will continue beyond the pandemic. According to the Canadian Labour Force Survey, 80% of employees who began working from home during COVID-19, indicated a preference to work half of their hours from home after the pandemic [5]. Similarly, a large survey across 25 countries reported that 90% of employees preferred to continue WFH in some capacity [6, 7]. Nearly 60% of employers expect either all or part of their workforce to continue working from home once the pandemic is over [8]. Based on these findings, it is likely that some form of remote work arrangement, such as hybrid WFH, might become the new normal, signifying the need for understanding the health and well-being of employees working from home.

Research has shown both benefits and challenges with voluntary and mandatory WFH. Before the pandemic, WFH was primarily offered on a voluntary or part-time basis. Workers who chose to WFH may experience lower work-related stress, higher quality of life due to increased autonomy, and greater overall well-being [9–12]. Despite the benefits, WFH employees may be unable to disengage from their work and may incorporate job duties into their family life, therefore negatively affecting their work-life balance [13]. Voluntary WFH improves personal and organizational productivity and performance; however, the positive effects are less pronounced in mandatory WFH arrangements [14]. Several cross-sectional studies have shown that mandatory WFH during the pandemic can result in lower mental and physical health and well-being [15], workplace comfort [16], physical activity [16] as well as greater low back pain [17] and stress [18] compared to pre-pandemic levels.

Despite the emerging research surrounding the health and well-being of WFH employees, there is still a need for assessing the within-individual changes in health and well-being of employees working from home over the course of the pandemic. A thorough understanding of the changes in mental and physical health and well-being of employees working from home during the pandemic allows organizations to develop targeted resources to better equip their workforce to adapt to remote work or inform their work arrangement policies. In this paper, we present the findings of two surveys (initial and 6-month follow-up) conducted in Canada to examine the health and well-being of employees who were working from home during the COVID-19 pandemic. We investigated the within-individual changes in mental and physical health and well-being of WFH employees who completed both the initial and follow-up surveys.

## Methods

### Participants

We recruited Canadian adults who were aged 18 years or older, and currently working or have worked from home at least two days per week during the COVID-19 pandemic. Students without part-time or full-time jobs were excluded from the study. Participants were recruited via advertisements on online social media platforms such as Facebook, LinkedIn, Reddit, and Twitter, both radio and print media, as well as snowball and purposive sampling using our networks such as unions, employer groups from various sectors, and health and safety associations. This study was approved by the Conestoga College’s Research Ethics Board.

### Survey

We conducted a panel study over two time periods. Two online surveys (initial and 6-month follow-up) were distributed nationwide to collect data on demographics, WFH preferences, employment situation, mental and physical health, workstation characteristics, and technology usage (see Additional Files 1 & 2 for the initial and follow-up survey, respectively). The initial survey was conducted from October 2020 to December 2020 during the second wave of COVID-19 in Canada [19]. Participants who completed the initial survey were invited to provide their contact information if they wished to take part in the 6-month follow-up survey, conducted from May 2021 to June 2021 during the end of the third wave of the pandemic in Canada [19]. The initial and follow-up surveys were distributed via Qualtrics, and each survey took approximately 20 min to complete. Participants provided informed consent before starting the surveys. Every question included a *Prefer not to say* response option for the participants.

We collected demographic data including age, gender, province of residence, living arrangements, as well as employment-related information such as type of industry, main role at work, and frequency of WFH. Nine employment-related questions involving employment status, salary changes, and sick days were included only in the follow-up survey. In both surveys, participants also responded to questions pertaining to their children and other dependents.

We measured psychosocial factors, general mental health, burnout, stress, and job satisfaction using the Copenhagen Psychosocial Questionnaire (COPSOQ) [20]. Psychosocial factors were measured based on 33 items from COPSOQ, all on 5-point Likert scales. Work-family conflict (i.e. work-related duties interfere with home/family responsibilities) and family-work conflict (i.e. home/family responsibilities interfere with work-related duties) were measured using ten items on a 7-point Likert scale [21]. We also included eight items using a 5-point Likert scale in both surveys to assess respondents’ WFH experience compared to their work situation either before the pandemic (in initial survey) or the previous six months (in follow-up survey).

Physical health was assessed through questions on sedentary behaviour and musculoskeletal pain/discomfort frequency and severity levels. Sedentary behaviour was measured using the Occupational Sitting and Physical Activity Questionnaire [22, 23]. Respondents rated their musculoskeletal discomfort/pain frequency and severity using a 5-point and 3-point Likert scale, respectively, in five body regions: neck and shoulders; hands and fingers; arms; middle and/or lower back; and hips, bottom, legs, or feet [24].

Respondents’ workstation comfort, workstation location, as well as hardware and software technology usage were also assessed. In both surveys, respondents indicated whether they received any training or guidance for setting up their workstations. In the initial survey, participants identified the hardware and software technologies provided by themselves or their employers. We measured technology support and productivity based on scales developed by Oakman and colleagues [25]. Technology complexity was solely assessed in the initial survey using two items from the Technostress Creators Scale, allowing us to measure technostress (i.e. stress due to the use of information and communication technologies) [26]. In the follow-up survey, we asked four additional productivity-related items on technology use.

Participants were asked their preferred number of days per week to WFH. In the follow-up survey, participants specified their reasons for preferring to either continue or not continue to WFH and indicated whether their organization is planning on returning to work in the next year.

### Analysis

Frequencies of responses were calculated as a percentage of the total sample size (Initial n = 1617; Follow-up n = 382) for survey questions involving demographics, WFH preferences, workstation setup training, employment situation, provision of hardware technologies, provision and usage of software technologies, and organization’s return to work plan. The number of participants who selected *Prefer not to say* as a response option are not presented in the frequency tables; therefore, the sum of proportions for certain questions may be less than 100% of the total sample size. Means and standard deviations (SD) were calculated for technology support, technology complexity, and productivity after removing participants who selected *Prefer not to say* as a response option.

To analyze the within-individual changes in mental and physical health and well-being of the 382 respondents who completed both the initial and follow-up surveys, we conducted a Wilcoxon signed-rank test separately for each of the following variables: COPSOQ dimensions, work-family conflict, family-work conflict, satisfaction with the division of household tasks and childcare duties, WFH experience, pain score for each body region, sedentary behaviour, and workstation comfort. Means and SD were calculated for each variable separately after excluding participants who selected *Prefer not to say* as a response option in either of the surveys. To determine the score of a COPSOQ dimension, we calculated the average score across all questions within the dimension. Pain score for each body region was calculated by multiplying the pain frequency and severity [24].

McNemar-Bowker tests were conducted to assess the within-individual changes in (a) workstation location and (b) the usage of separate keyboard and/or mouse with laptop over time. We also conducted McNemar’s tests to examine the within-individual changes in the usage of the following hardware technologies: adjustable chair, laptop, secondary monitor with laptop, desktop, secondary screen with desktop, and phone/tablet. Frequencies of responses were calculated as a percentage of the number of participants who completed both surveys, separately for workstation location and each hardware technology. Contingency tables for McNemar-Bowker and McNemar’s tests are presented in Additional File 3.

Participants who selected *Prefer not to say* as a response option in either of the surveys were removed before conducting each statistical test separately; therefore, the sample size for certain tests may be fewer than 382. Statistical analyses were conducted using R version 4.1.2 with α = 0.05.

## Results

We present survey findings in four sections. The first section presents the demographic information and surveyed responses to the number of preferred WFH days and workstation set up training of both the initial (n = 1617) and follow-up (n = 382) surveys.. The following two sections report the results of the questions that were asked exclusively in the initial (n = 1617) and follow-up (n = 382) surveys.. The final section presents the results of the within-individual analyses of the mental and physical health and well-being measures, workstation comfort, workstation location, and hardware technology usage of the participants who completed both surveys.

### Results of questions asked in the initial (n = 1617) and follow-up (n = 382) surveys

#### Demographics

A total of 1617 participants completed the initial survey, of which 382 respondents completed the follow-up survey. Majority of the participants lived in Ontario (Initial = 79.4%; Follow-up = 85.3%) and over 68% of the total respondents were women (Table 1).

More than half of participants in both surveys worked in the public sector and in various industries such as *Education and Training*; *Professional, Scientific, and Technical Services*; *Public Administration and Safety*; and *Information, Media, and Telecommunications* (Table 1). Approximately 15% of the respondents worked as *Managers*, whereas most of the remaining participants identified themselves as *Professional* or *Clerical/Administrative Worker*. More than 75% of participants worked in large organizations with over 200 employees.

According to both surveys, most respondents lived with one or more adults (Table 1). More than 30% of the participants lived with children, of which most of them had their children at home during work hours. Less than 20% of respondents had dependents other than children.

**Table 1:** Demographics of participants for initial and follow-up surveys

	Initial (n = 1617)	Follow-up (n = 382)
**Age** ^**b**^		
18–25 years	37 (2.3%)	10 (2.6%)
26–35 years	377 (23.3%)	114 (29.8%)
36–45 years	439 (27.1%)	102 (26.7%)
46–55 years	428 (26.5%)	86 (22.5%)
56 years and over	336 (20.8%)	69 (18.1%)
**Gender** ^**b**^		
Man	509 (31.5%)	115 (30.1%)
Woman	1108 (68.5%)	262 (68.6%)
Non-binary	NA ^**e**^	3 (0.8%)
Other	0 (0.00%)	NA ^**e**^
**Province**		
Alberta	24 (1.5%)	6 (1.6%)
British Columbia	70 (4.3%)	17 (4.5%)
Manitoba	45 (2.8%)	8 (2.1%)
New Brunswick	65 (4%)	6 (1.6%)
Newfoundland & Labrador	32 (2%)	6 (1.6%)
Nova Scotia	11 (0.7%)	5 (1.3%)
Nunavut	12 (0.7%)	2 (0.5%)
Ontario	1284 (79.4%)	326 (85.3%)
Prince Edward Island	2 (0.1%)	0 (0.0%)
Quebec	23 (1.4%)	1 (0.3%)
Saskatchewan	48 (3%)	5 (1.3%)
Yukon	1 (0.1%)	0 (0.0%)
**Industry** ^**a**^		
Accommodation and Food Services	3 (0.2%)	1 (0.3%)
Agriculture, Forestry, Fishing	7 (0.4%)	2 (0.5%)
Arts, Recreation Services	12 (0.7%)	4 (1%)
Construction	53 (3.3%)	11 (2.9%)
Education and Training	285 (17.6%)	91 (23.8%)
Electricity, Gas, Water and Waste Services	28 (1.7%)	7 (1.8%)
Financial and Insurance Services	153 (9.5%)	28 (7.3%)
Healthcare and Social Assistance	178 (11%)	33 (8.6%)
Information, Media and Telecommunications	207 (12.8%)	40 (10.5%)
Manufacturing	65 (4%)	16 (4.2%)
Mining	5 (0.3%)	3 (0.8%)
Other Services	10 (0.6%)	2 (0.5%)
Professional, Scientific, and Technical Services	240 (14.8%)	52 (13.6%)
Public Administration and Safety	222 (13.7%)	31 (8.1%)
Rental, Hiring & Real Estate Services	5 (0.3%)	2 (0.5%)
Retail Trade	11 (0.7%)	2 (0.5%)
Transport, Postal & Warehousing	22 (1.4%)	9 (2.4%)
Wholesale Trade	6 (0.4%)	2 (0.5%)
Other	78 (4.8%)	46 (12%)
**Sector** ^**b**^		
Not for profit sector	281 (17.4%)	62 (16.2%)
Private sector	508 (31.4%)	115 (30.1%)
Public sector	814 (50.3%)	197 (51.6%)
Self employed	14 (0.9%)	3 (0.8%)
**Role** ^**b**^		
Clerical/Administrative Worker	583 (36.1%)	113 (29.6%)
Community & Personal Service Worker	21 (1.3%)	8 (2.1%)
Labourers	5 (0.3%)	1 (0.3%)
Machinery Operators & Drivers	3 (0.2%)	1 (0.3%)
Manager	238 (14.7%)	62 (16.2%)
Professional	680 (42.1%)	168 (44%)
Sales Worker	37 (2.3%)	11 (2.9%)
Technician & Trade Worker	50 (3.1%)	15 (3.9%)
**Business size** ^**b**^		
Sole Trader/Self-Employed	12 (0.7%)	2 (0.5%)
2–19 employees	128 (7.9%)	30 (7.9%)
20–199 employees	234 (14.5%)	47 (12.3%)
200 + employees	1243 (76.9%)	301 (78.8%)
**Living arrangements**		
Alone	195 (12.1%)	45 (11.8%)
With one or more adults	825 (51%)	212 (55.5%)
With one or more adults and children below 18 years	550 (34%)	117 (30.6%)
With one or more children below 18 years	47 (2.9%)	8 (2.1%)
**Number of children receive care** ^**a, c**^		
None	32 (2%)	6 (1.6%)
1	218 (13.5%)	43 (11.3%)
2	274 (16.9%)	63 (16.5%)
3	55 (3.4%)	11 (2.9%)
4	12 (0.7%)	2 (0.5%)
5 or more	3 (0.2%)	0 (0.0%)
**Number of children at home when WFH** ^**c**^		
None	170 (10.5%)	11 (2.9%)
1	223 (13.8%)	50 (13.1%)
2	165 (10.2%)	53 (13.9%)
3	31 (1.9%)	9 (2.4%)
4	5 (0.3%)	2 (0.5%)
5 or more	3 (0.2%)	0 (0.0%)
**Age group(s) of children at home when WFH** ^**c, d**^		
Less than 4 years	106 (6.6%)	23 (6%)
4–6 years	86 (5.3%)	26 (6.8%)
6–8 years	65 (4%)	35 (9.2%)
8–12 years	116 (7.2%)	39 (10.2%)
12–16 years	127 (7.9%)	34 (8.9%)
16–18 years	80 (4.9%)	22 (5.8%)
**Homeschooling children** ^**a, b, c**^		
No	513 (31.7%)	96 (25.1%)
Yes	78 (4.8%)	26 (6.8%)
**Number of hours involved in children’s education** ^**a, c**^		
None	6 (0.4%)	2 (0.5%)
1–2 h	32 (2%)	12 (3.1%)
2–3 h	15 (0.9%)	5 (1.3%)
3–4 h	11 (0.7%)	3 (0.8%)
More than 4 h	9 (0.6%)	4 (1%)
**Number of days per week children attend virtual classes** ^**c**^		
1 day	11 (0.7%)	3 (0.8%)
2 days	8 (0.5%)	1 (0.3%)
3 days	8 (0.5%)	2 (0.5%)
4 days	6 (0.4%)	0 (0.0%)
5 days	46 (2.8%)	19 (5%)
**Dependents other than children**		
No	1345 (83.2%)	329 (86.1%)
Yes, adult(s) living elsewhere	108 (6.7%)	32 (8.4%)
Yes, adult(s) living with me	135 (8.3%)	17 (4.5%)
Other	19 (1.2%)	1 (0.3%)

^a^ The number of respondents who chose *Prefer not to say* as the response option in the initial survey are not presented; therefore, the sum of proportions is less than 100%

^b^ The number of respondents who chose *Prefer not to say* as the response option in the follow-up survey are not presented; therefore, the sum of proportions is less than 100%

^c^ Only respondents who had children answered the question; therefore, the sum of proportions is less than 100%

^d^ Sum of proportions is not equal to 100% because participants might have chosen more than one response

^e^*Non-binary* was included as a response option only in the follow-up survey, whereas *Other* was a response option included only in the initial survey

#### WFH preferences: number of preferred days

Based on both surveys, most respondents preferred to WFH for three or more days per week regardless of the pandemic situation and risk level (Table 2).

**Table 2:** WFH preferences of respondents

	Initial (n = 1617)	Follow-up (n = 382)
**Number of days prefer to WFH in non-pandemic time** ^**b**^		
None	105 (6.5%)	24 (6.3%)
1 day/week	84 (5.2%)	19 (5%)
2 days/week	275 (17%)	68 (17.8%)
3 days/week	360 (22.3%)	105 (27.5%)
4 days/week	210 (13%)	45 (11.8%)
Every day	583 (36.1%)	119 (31.2%)
**Number of days prefer to WFH with low risk of COVID-19** ^**a, b**^		
None	95 (5.9%)	19 (5%)
1 day/week	60 (3.7%)	16 (4.2%)
2 days/week	191 (11.8%)	61 (16%)
3 days/week	266 (16.5%)	90 (23.6%)
4 days/week	226 (14%)	51 (13.4%)
Every day	769 (47.6%)	141 (36.9%)
**Number of days prefer to WFH with high risk of COVID-19** ^**a, b**^		
None	91 (5.6%)	21 (5.5%)
1 day/week	7 (0.4%)	4 (1%)
2 days/week	28 (1.7%)	4 (1%)
3 days/week	42 (2.6%)	20 (5.2%)
4 days/week	63 (3.9%)	9 (2.4%)
Every day	1376 (85.1%)	322 (84.3%)

^a^ The number of respondents who chose *Prefer not to say* as the response option in the initial survey are not presented; therefore, the sum of proportions is less than 100%

^b^ The number of respondents who chose *Prefer not to say* as the response option in the follow-up survey are not presented; therefore, the sum of proportions is less than 100%

#### Workstation setup training

In the initial survey, 53.1% (n = 859) of the participants received suggestions about workstation setup from their employers when they started to WFH. At follow-up, only 30.9% (n = 118) of the respondents had received additional training.

### Results of questions asked only in the initial survey (n = 1617)

#### Employment situation: frequency of WFH

Based on the initial survey, more than 75% of participants did not work from home before COVID-19 (Supplementary Table 1 in Additional File 4). During the pandemic, 87% of the participants worked from home for 5 or more days per week. More than 90% of respondents worked from home on a full-time basis. On average, respondents were working from home for approximately eight months since the start of the pandemic.

#### Provision of hardware technology

Many of the participants indicated that they received a laptop (67.8%), mouse (61.3%), or keyboard (45.1%) from their employers during WFH (Supplementary Table 2 in Additional File 4). Some of the respondents provided themselves with their own equipment (laptops = 16.5%; mouse = 29.4%; keyboard = 19.9%).

#### Provision and usage of software technology

Most of the respondents were supplied with online meeting platforms, access to organization network, and work-related software programs (Supplementary Table 2 in Additional File 4). Nearly all participants used video conferencing software in the initial survey. More than 60% of the respondents spent less than two hours on telephone or video conferencing.

#### Technology support, technology complexity, and productivity

In the initial survey, participants generally agreed that they received good support if they encountered technology problems during work (technology support score = 3.87 ± 0.78; n = 1600). On average, respondents found it not difficult to learn how to use new technologies (technology complexity score = 2.49 ± 0.99; n = 1604). Participants primarily agreed that they can work effectively using their hardware or software technology (productivity score = 4.29 ± 0.79; n = 1602).

### Results of questions asked only in the follow-up survey (n = 382)

#### Employment situation: employment status, salary changes, sick days, and working arrangements

Based on the follow-up survey, nearly all participants were employed and had not commenced new employment within the last six months (Supplementary Table 3 in Additional File 4). A small proportion (2.9%) of participants experienced a decrease in their salary. Furthermore, only 9% of respondents used more vacation or sick days than before the pandemic, and 85% of participants were satisfied with the amount of flexibility and sick days available to them. Over 80% of the respondents continued to WFH the entire time in their past six months.

#### Productivity

Participants primarily agreed that they can work effectively using their hardware or software technology in the follow-up survey (productivity score = 3.63 ± 0.47; n = 376).

#### WFH preferences: primary reasons for preference

In the follow-up survey, we asked participants about their primary reasons for their WFH preferences. The most common responses among the participants who preferred to WFH were flexible work environment (28.8%), better work-life balance (17.5%), reduced commuting time and costs (18.6%), and higher productivity (16.8%) (Supplementary Table 4 in Additional File 4).

#### Organization’s return to work plan

According to the follow-up survey, only 13% of the participants stated that their organizations were planning to return to work on a full-time basis in the next year (Supplementary Table 4 in Additional File 4). Nearly 40% would be either returning to the office on a part-time basis, allowed to choose their work location, or working from home full-time. The remaining respondents (47%) were not aware of their organization’s plan on returning to the office in the upcoming year.

### Results of within-individual analyses between initial and follow-up

#### Mental and physical health and well-being

Our within-individual analyses of the participants who completed both surveys showed significant changes in various aspects of their mental health and well-being over time. Burnout, stress, general mental health, and job insecurity levels were significantly higher during initial survey compared to follow-up (Table 3). Additionally, participants reported that they (a) were provided with more information that keeps them in touch with workplace events and developments, (b) received more help and feedback from colleagues, and (c) experienced a better sense of community with their colleagues over time (Table 3).

Physical health and well-being of participants who completed both surveys varied between the two time points. Neck and shoulder pain scores were significantly higher during the initial survey compared to the follow-up (Table 3). Furthermore, the average proportion of time spent sitting during work hours decreased by 1.5% whereas the percentage of time spent on heavy labour or physically demanding tasks during non-work hours increased by 1.2% over time (Table 3). On average, participants spent more than 80% of their work time sitting over both time points.

**Table 3:** Mental and physical health and well-being measures of participants who completed both the initial and follow-up surveys

	Total (n)	Initial (Mean ± SD)	Follow-up (Mean ± SD)	p-value
**COPSOQ dimensions (max score = 5)**				
Quantitative Demands ^a^	382	2.47 ± 0.97	2.53 ± 0.98	0.236
Influence at Work ^a^	371	3.11 ± 1.02	3.10 ± 1.00	0.652
Predictability ^b^	377	3.31 ± 0.99	3.33 ± 0.98	0.904
Recognition ^b^	366	3.87 ± 1.06	3.81 ± 1.09	0.167
Role Clarity ^b^	378	3.91 ± 0.92	3.91 ± 0.85	0.614
Role Conflict ^b^	372	2.69 ± 1.12	2.65 ± 1.05	0.633
Quality of Leadership ^b^	306	3.54 ± 1.17	3.47 ± 1.16	0.206
Social Support from Supervisor ^a^	366	4.11 ± 1.05	4.05 ± 1.02	0.211
Social Support from Colleagues ^a^	371	4.08 ± 0.90	4.07 ± 0.91	0.961
Sense of Community at Work ^a^	377	3.97 ± 0.87	3.90 ± 0.86	0.076
Job Insecurity ^b^	334	2.64 ± 1.23	2.44 ± 1.17	**< 0.001**
Insecurity over Working Conditions ^b^	292	1.84 ± 1.00	1.66 ± 0.91	**0.002**
Job Satisfaction ^d^	381	3.97 ± 1.09	4.01 ± 0.98	0.604
Vertical Trust ^b^	352	3.73 ± 1.00	3.69 ± 0.96	0.222
Organizational Justice ^b^	280	3.44 ± 1.06	3.51 ± 1.02	0.217
Burnout ^a^	382	3.31 ± 0.90	3.19 ± 0.98	**0.001**
Stress ^a^	382	3.09 ± 0.89	2.97 ± 0.98	**0.001**
Somatic Stress ^a^	381	2.27 ± 0.84	2.20 ± 0.88	0.098
Cognitive Stress ^a^	382	2.64 ± 0.87	2.68 ± 0.98	0.328
General Physical Health ^c^	382	3.11 ± 0.95	3.07 ± 0.91	0.281
General Mental Health ^c^	382	3.08 ± 0.99	2.91 ± 0.95	**< 0.001**
**Work-family and family-work conflicts (max score = 7)**				
Work-family conflict ^e^	382	3.25 ± 1.70	3.18 ± 1.64	0.168
Family-work conflict ^e^	382	2.58 ± 1.54	2.65 ± 1.55	0.275
**Satisfaction with division of childcare and household tasks (max score = 5)**				
Childcare tasks ^f^	75	3.81 ± 1.17	3.60 ± 1.22	0.125
Household tasks ^f^	332	3.68 ± 1.20	3.64 ± 1.20	0.515
**WFH experience** ^**g**^ **(max score = 5)**				
I can get help and feedback from my work colleagues, if needed ^h^	375	2.76 ± 0.83	3.00 ± 0.71	**< 0.001**
I can get help and feedback from my immediate supervisor, if needed ^h^	370	2.89 ± 0.81	2.98 ± 0.78	0.095
I receive information that keeps me in touch with workplace events and developments ^h^	376	2.88 ± 0.87	2.99 ± 0.77	**0.026**
I feel a good sense of community with my work colleagues ^h^	376	2.54 ± 1.03	2.82 ± 0.83	**< 0.001**
Trying to work productively is stressful or frustrating ^h^	375	3.07 ± 1.17	3.01 ± 0.95	0.290
Work interferes with my home or family life ^h^	375	2.89 ± 1.14	2.90 ± 0.96	0.767
I often feel tired or exhausted ^h^	377	3.27 ± 1.18	3.28 ± 1.01	0.764
I enjoy my work and the job overall ^h^	376	3.15 ± 1.05	3.10 ± 0.93	0.299
**Pain score (max score = 12)**				
Neck and shoulders ^i^	379	3.70 ± 3.15	3.33 ± 2.96	**0.004**
Hands and fingers ^i^	381	1.46 ± 2.25	1.51 ± 2.34	0.581
Arms ^i^	381	1.07 ± 2.01	0.96 ± 1.92	0.204
Middle and/or lower back ^i^	381	3.17 ± 3.15	3.18 ± 3.31	0.512
Hips, bottom, legs, or feet ^i^	381	2.19 ± 2.80	2.43 ± 3.12	0.402
**Sedentary behaviour (% of time)**				
During work hours				
Sitting	382	84.37 ± 17.88	82.90 ± 19.48	**0.035**
Standing	382	8.66 ± 11.98	9.67 ± 13.57	0.196
Walking	382	5.91 ± 7.24	5.95 ± 7.51	0.831
Performing heavy labour or physically demanding tasks	382	0.40 ± 2.85	0.43 ± 2.39	0.340
During non-work hours				
Sitting	382	50.35 ± 24.75	48.70 ± 23.08	0.178
Standing	382	22.82 ± 16.77	21.34 ± 14.41	0.108
Walking	382	21.12 ± 15.05	22.27 ± 14.87	0.332
Performing heavy labour or physically demanding tasks	382	4.28 ± 8.16	5.44 ± 8.45	**0.014**

^a^ Measured on a 5-point scale from *never* (1) to *always* (5)

^b^ Measured on a 5-point scale from *to a very small extent* (1) to *to a very large extent* (5)

^c^ Measured on a 5-point scale from *poor* (1) to *excellent* (5)

^d^ Measured on a 5-point scale from *very unsatisfied* (1) to *very satisfied* (5)

^e^ Measured on a 7-point scale from *strongly disagree* (1) to *strongly agree* (7)

^f^ Measured on a 5-point scale from *very dissatisfied* (1) to *very satisfied* (5).

^g^ In initial survey, participants compared their WFH experience to their pre-pandemic work situation. In follow-up survey, they compared their WFH experience to their work situation six months ago.

^h^ Measured on a 5-point scale from *much less than before* (1) to *much more than before* (5).

^i^ Pain score was calculated by multiplying the pain freqWorkstation comfort, workstation location, and hardware technology usage.

Workstation comfort significantly changed over time (p < 0.001). The average workstation comfort rating was higher during the follow-up survey (3.06 ± 1.27; n = 381) compared to the initial survey (2.86 ± 1.30; n = 381).

There was a significant change in the usage of adjustable chairs (χ^2^ = 5.97; p = 0.015) and separate monitor with laptop (χ^2^ = 5.30; p = 0.021) over time (Table 4). A total of 44 (11.5%) respondents started using adjustable chairs, and 26 (6.8%) participants began using separate monitors with their laptops over time (see Supplementary Tables 2 & 5 in Additional File 3 for contingency tables).

**Table 4:** Workstation location and hardware technology usage of respondents who completed both initial and follow-up surveys

	Total (n)	Initial	Follow-up	p-value
**Workstation location**	382			0.055 ^a^
Separate Room		252 (66%)	266 (69.6%)	
Separate Room with interruptions		65 (17%)	68 (17.8%)	
Work Wherever		65 (17%)	48 (12.6%)	
**Hardware technology usage**				
Adjustable chair	381			**0.015** ^b^
No		113 (29.7%)	92 (24.1%)	
Yes		268 (70.3%)	289 (75.9%)	
Laptop	382			0.579 ^b^
No		43 (11.3%)	40 (10.5%)	
Yes		339 (88.7%)	342 (89.5%)	
Separate keyboard and/or mouse with laptop	376			0.136 ^a^
Both keyboard and mouse		234 (62.2%)	248 (66%)	
Keyboard only		2 (0.5%)	4 (1.1%)	
Mouse only		63 (16.8%)	63 (16.8%)	
Neither		77 (20.5%)	61 (16.2%)	
Secondary monitor with laptop	335			**0.021** ^b^
No		112 (33.4%)	97 (29%)	
Yes		223 (66.6%)	238 (71%)	
Desktop	382			0.409 ^b^
No		235 (61.5%)	243 (63.6%)	
Yes		147 (38.5%)	139 (36.4%)	
Secondary screen with desktop	107			1 ^b^
No		35 (32.7%)	35 (32.7%)	
Yes		72 (67.3%)	72 (67.3%)	
Phone/Tablet	382			0.374 ^b^
No		79 (20.7%)	87 (22.8%)	
Yes		303 (79.3%)	295 (77.2%)	

^a^ McNemar-Bowker test was conducted to determine significant differences in the responses of multiple-choice questions (e.g. workstation location) over time

^b^ McNemar’s test was conducted to determine significant differences in the responses of dichotomous questions (e.g. usage of adjustable chair) over time

## Discussion

We examined the changes in mental and physical health and well-being of employees who were working from home in Canada during the COVID-19 pandemic by comparing the findings of the initial and follow-up surveys. We identified significant decreases in burnout, stress, general mental health, and job insecurity levels over our two reporting periods, six months apart. Respondents in both surveys reported that 80% of their work time was spent sitting despite a reduction in work-related sedentary behaviour over time.

Based on our analysis of the 382 respondents who completed both initial and follow-up surveys, we observed lower burnout and stress levels; however, their self-reported general mental health declined over time. The decreased levels of burnout and stress may be due to greater co-worker support received by the respondents [27, 28], which was supported by our survey findings, where participants reported that they received more help and feedback from their colleagues and experienced a better sense of community with their co-workers over the course of the pandemic (Table 3). Despite the reduction in burnout and stress levels, self-reported general mental health decreased over time. Emerging research have shown that mental well-being of WFH employees could be affected by demographic variables such as age [15, 18], gender [15, 18, 29], presence of children [15, 29], type of industry [15], as well as socioeconomic variables such as employment status [30], education level [31], and economic status [32]. However, many of these demographic and socioeconomic variables were similar between our two reporting periods. Instead, the decline in general mental health may be due to frequent exposure to pandemic-related news [31, 33–35]. During the follow-up survey period (May to June 2021), a higher number of patients were hospitalized and admitted to intensive care units due to COVID-19 compared to the initial survey period (October to December 2020) [19]. Moreover, in the follow-up period, WHO declared a new variant of COVID-19 known as the Delta variant, and the number of reported cases was rising rapidly across Canada [36]. As a result, participants might have been more worried about their family members, friends, or themselves contracting COVID-19, thus negatively impacting their mental health [32].

Levels of job insecurity reduced between the two reporting periods. During the initial survey, participants might have felt more insecure about their employment due to exposure to news regarding the increase in unemployment rates [37] resulting from layoffs [38] and closures of businesses [39] due to the pandemic [40, 41]. In the follow-up survey, nearly all participants reported that they were employed in the past six months. Due to the stable employment status over time, respondents likely felt less insecure about their jobs, resulting in the decreased levels of job insecurity at follow-up.

Since the start of the pandemic, sedentary behaviour among WFH employees has generally increased [42–44]. However, in our study, the average percentage of time spent sitting during work hours decreased by 1.5% whereas the average proportion of time spent on heavy labour or physically demanding tasks during non-work hours increased by 1.2% over time. This finding suggests that respondents may have been modifying their lifestyle to reduce their sedentary behaviour. Perhaps, over time, the participants started engaging in home-based physical activities due to the pandemic-related restrictions such as closures of fitness facilities and gymnasiums during the study periods. Furthermore, poorer climate during the fall and early winter season in Canada may have resulted in greater sedentary behaviour in the initial survey (October to December) compared to the follow-up period (May to June), where participants likely had greater opportunities to engage in outdoor physical activities. Despite the reduction in sedentary behaviour, the average proportion of time spent sitting during work hours was more than 80% in both surveys. This finding is concerning because prolonged sedentary hours can increase the risk of poor physical health outcomes such as obesity, cancer, as well as cardiovascular and metabolic diseases [45]. Due to WFH arrangements and pandemic-related restrictions, participants may have had limited opportunities to engage in work-related physical activities, such as commuting to work and going out for lunch breaks [42, 46], thus contributing to the high sedentary behaviour during work hours.

WFH is likely to continue in the future. Based on our findings, nearly 70% of the respondents who began working from home during COVID-19 preferred to WFH for three or more days per week during a non-pandemic time. This finding is consistent with a recent Canadian Labour Force Survey that showed 80% of employees who started to WFH since the pandemic preferred to work at least half of their hours from home after the pandemic [5]. Most participants in our study preferred to continue to WFH due to the flexible work environment, better work-life balance, lower commuting cost and time, as well as higher productivity. Accordingly, organizations will need to consider their policies and procedures to adequately support WFH, whilst protecting their employees’ health and well-being.

There are a few limitations in our study. First, there could have been a potential for recall bias among the participants when responding to certain questions that required them to recall the last six months or the period prior to the pandemic. Second, mental and physical health and well-being outcomes could be affected by seasonal variations [47, 48] and pandemic-related restrictions [49–51]; therefore, we caution the interpretation of our findings. Third, there is a potential for selection bias because our study included respondents who were primarily women, lived in Ontario, worked in non-manager roles, and were employed in large organizations; thus, the study sample may not be representative of the general population. Fourth, the scope of our study was to examine the within-individual differences in mental and physical health and well-being of Canadian employees who were working from home between two time points during the pandemic. The design of our study allowed us to investigate the changes in health and well-being of the same group of WFH employees over the course of the pandemic. However, we note that a limitation of our study is the absence of participants who were not working from home during the pandemic; therefore, we could not compare our current findings to a reference group. That said, in a future paper, we will be examining the effects of WFH arrangements on mental and physical health and well-being by comparing our findings to population-based reference values according to COPSOQ. Finally, of the 1617 participants who completed the initial survey, only 382 completed the follow-up survey; hence, the high dropout rate in our study may have affected the interpretation of our findings such as the reduction in job insecurity levels. Panel attrition is a concern in longitudinal surveys because it may bias the survey findings if the respondents who dropped out are systematically different from those who stayed in the study [52]. Gender has been shown to be strongly associated with panel attrition [53]; after conducting a chi-squared analysis, we found no significant relationship between gender and the attrition status of participants in our study. This finding suggests that panel attrition may not have significantly biased our findings. Despite the high dropout rate, our study provided insights into the within-individual changes in mental and physical health and well-being of employees who were working from home over the course of the pandemic in Canada.

## Conclusion

WFH employees experienced significant changes in certain aspects of their mental and physical health during the COVID-19 pandemic. Burnout, stress, general mental health, and job insecurity levels decreased over time. Average proportion of work time spent sitting was high (> 80%) in both surveys despite a significant decrease in self-reported work-related sedentary behaviour. Participants also reported that they experienced a better sense of community with their co-workers and received more help and feedback from their colleagues over time. The findings of our study can inform organizations and employees on the changes in health and well-being of employees working from home during the pandemic. By understanding the changes in health and well-being, employers can create better strategies and implement organizational policies that help improve workers’ health and well-being.

## Electronic supplementary material

Below is the link to the electronic supplementary material.

Supplementary Material 1

Supplementary Material 2

Supplementary Material 3

Supplementary Material 4

## Data Availability

The datasets generated during and/or analysed during the current study are available from the corresponding author on reasonable request.

## List of abbreviations

COPSOQ: Copenhagen Psychosocial Questionnaire
WFH: Work from home

## References

[1] Statistics Canada. Table 1: Percentage of workers working from home, by selected characteristics, April 2020 to June 2021. 2021. https://www150.statcan.gc.ca/n1/daily-quotidien/210804/t001b-eng.htm. Accessed 4 Apr 2022.

[2] Beck MJ, Hensher DA. Working from home in Australia in 2020: Positives, negatives and the potential for future benefits to transport and society. 2021. 10.1016/j.tra.2022.03.016 PMC9759339 36568131

[3] Eurofound. Working during COVID-19. 2020. https://www.eurofound.europa.eu/data/covid-19/working-teleworking%0A. Accessed 4 Apr 2022.

[4] Statista. Change in remote work trends due to COVID-19 in the United States in 2020. 2020. https://www.statista.com/statistics/1122987/change-in-remote-work-trends-after-covid-in-usa/. Accessed 4 Apr 2022.

[5] Statistics Canada. Study: Working from home: Productivity and preferences. 2021. https://www150.statcan.gc.ca/n1/daily-quotidien/210401/dq210401b-eng.htm. Accessed 12 Apr 2022.

[6] Criscuolo C, Gal P, Leidecker T, Losma L, Nicoletti G. The role of telework for productivity during and post-COVID-19: Results from an OECD survey among managers and workers. 2021.

[7] Mehdi T, Morissette R. Working from home in Canada: What have we learned so far? 2021. https://www150.statcan.gc.ca/n1/pub/36-28-0001/2021010/article/00001-eng.htm. Accessed 4 Apr 2022.

[8] Statistics Canada. Percentage of workforce teleworking or working remotely, and percentage of workforce anticipated to continue primarily teleworking or working remotely after the pandemic, by business characteristics. 2020. https://www150.statcan.gc.ca/t1/tbl1/en/tv.action?pid=3310027401&pickMembers%5B0%5D=3.2. Accessed 4 Apr 2022.

[9] Anderson AJ, Kaplan SA, Vega RP (2015). The impact of telework on emotional experience: When, and for whom, does telework improve daily affective well-being?. Eur J Work Organizational Psychol.

[10] Filardi F, De Castro RMP, Zanini MTF (2020). Advantages and disadvantages of teleworking in Brazilian public administration: Analysis of SERPRO and Federal Revenue experiences. Cadernos EBAPEBR.

[11] Hornung S, Glaser J (2009). Home-based telecommuting and quality of life: Further evidence on an employee-oriented human resource practice. Psychol Rep.

[12] Oakman J, Kinsman N, Stuckey R, Graham M, Weale V (2020). A rapid review of mental and physical health effects of working at home: How do we optimise health?. BMC Public Health.

[13] Eddleston KA, Mulki J (2017). Toward Understanding Remote Workers’ Management of Work–Family Boundaries: The Complexity of Workplace Embeddedness. Group Organ Manag.

[14] Hackney A, Yung M, Somasundram KG, Oakman J, Nowrouzi-Kia B, Yazdani A. Working in the digital economy: A systematic review of the impact of work from home arrangements on personal and organizational performance and productivity. PLOS ONE. Forthcoming 2022. 10.1371/journal.pone.0274728 PMC9555618 36223418

[15] Xiao Y, Becerik-Gerber B, Lucas G, Roll SC (2021). Impacts of working from home during COVID-19 pandemic on physical and mental well-being of office workstation users. J Occup Environ Med.

[16] Argus M, Pääsuke M (2021). Effects of the COVID-19 lockdown on musculoskeletal pain, physical activity, and work environment in Estonian office workers transitioning to working from home. Work.

[17] Guler MA, Guler K, Guneser Gulec M, Ozdoglar E (2021). Working from home during a pandemic: Investigation of the impact of COVID-19 on employee health and productivity. J Occup Environ Med.

[18] Hayes SW, Priestley JL, Moore BA, Ray HE. Perceived stress, work-related burnout, and working from home before and during COVID-19: An examination of workers in the United States. Sage Open. 2021;11.

[19] Government of Canada. COVID-19 daily epidemiology update. 2022. https://health-infobase.canada.ca/covid-19/epidemiological-summary-covid-19-cases.html?stat=num&measure=total&map=pt#a2%0A. Accessed 4 Apr 2022.

[20] Burr H, Berthelsen H, Moncada S, Nübling M, Dupret E, Demiral Y (2019). The Third Version of the Copenhagen Psychosocial Questionnaire. Saf Health Work.

[21] Netemeyer RG, Boles JS, McMurrian R (1996). Development and validation of work-family conflict and family-work conflict scales. J Appl Psychol.

[22] Chau JY, van der Ploeg HP, Dunn S, Kurko J, Bauman AE (2012). Validity of the occupational sitting and physical activity questionnaire. Med Sci Sports Exerc.

[23] Dillon K, Hiemstra M, Mitchell M, Bartmann N, Rollo S, Gardiner PA, et al. Validity of the occupational sitting and physical activity questionnaire (OSPAQ) for home-based office workers during the COVID-19 global pandemic: A secondary analysis. Appl Ergon. 2021;97. 10.1016/j.apergo.2021.103551 PMC9746924 34403840

[24] Oakman J, Macdonald W, Wells Y (2014). Developing a comprehensive approach to risk management of musculoskeletal disorders in non-nursing health care sector employees. Appl Ergon.

[25] Oakman J, Kinsman N, Lambert K, Stuckey R, Graham M, Weale V. Working from home in Australia during the COVID-19 pandemic: Cross-sectional results from the Employees Working From Home (EWFH) study. BMJ Open. 2022;12. 10.1136/bmjopen-2021-052733 PMC8980729 35379616

[26] Molino M, Ingusci E, Signore F, Manuti A, Giancaspro ML, Russo V (2020). Wellbeing costs of technology use during Covid-19 remote working: An investigation using the Italian translation of the technostress creators scale. Sustainability.

[27] Halbesleben JRB, Buckley MR (2004). Burnout in organizational life. J Manage.

[28] Sloan MM. Unfair treatment in the workplace and worker well-being: The role of coworker support in a service work environment. 2012.

[29] Graham M, Weale V, Lambert KA, Kinsman N, Stuckey R, Oakman J (2021). Working at home: The impacts of COVID 19 on health, family-work-life conflict, gender, and parental responsibilities. J Occup Environ Med.

[30] Mazza C, Ricci E, Biondi S, Colasanti M, Ferracuti S, Napoli C, et al. A nationwide survey of psychological distress among Italian people during the COVID-19 pandemic: Immediate psychological responses and associated factors. Int J Environ Res Public Health. 2020;17. 10.3390/ijerph17093165 PMC7246819 32370116

[31] Gao J, Zheng P, Jia Y, Chen H, Mao Y, Chen S, et al. Mental health problems and social media exposure during COVID-19 outbreak. PLOS ONE. 2020;15. 10.1371/journal.pone.0231924 PMC7162477 32298385

[32] Lei L, Huang X, Zhang S, Yang J, Yang L, Xu M. Comparison of prevalence and associated factors of anxiety and depression among people affected by versus people unaffected by quarantine during the COVID-19 epidemic in southwestern China. Medical Science Monitor. 2020;26. 10.12659/MSM.924609 PMC7199435 32335579

[33] Moghanibashi-Mansourieh A. Assessing the anxiety level of Iranian general population during COVID-19 outbreak. Asian J Psychiatr. 2020;51. 10.1016/j.ajp.2020.102076 PMC7165107 32334409

[34] Olagoke AA, Olagoke OO, Hughes AM (2020). Exposure to coronavirus news on mainstream media: The role of risk perceptions and depression. Br J Health Psychol.

[35] Xiong J, Lipsitz O, Nasri F, Lui LMW, Gill H, Phan L, et al. Impact of COVID-19 pandemic on mental health in the general population: A systematic review. J Affect Disord. 2020;:55–64. 10.1016/j.jad.2020.08.001 PMC7413844 32799105

[36] National Collaborating Centre for Infectious Diseases. Updates on COVID-19 variants of concern (VOC). 2022. https://nccid.ca/covid-19-variants/. Accessed 8 May 2022.

[37] Statistics Canada. Labour force characteristics, monthly, seasonally adjusted and trend-cycle, last 5 months. 2022. https://www150.statcan.gc.ca/t1/tbl1/en/tv.action?pid=1410028701. Accessed 8 May 2022.

[38] Statistics Canada. Layoffs since the start of the COVID-19 pandemic, by business characteristics. 2020. https://www150.statcan.gc.ca/t1/tbl1/en/tv.action?pid=3310027901. Accessed 8 May 2022.

[39] Statistics Canada. Monthly business openings and closures, Canada, January 2019 to June 2021, seasonally adjusted series. 2021. https://www150.statcan.gc.ca/n1/daily-quotidien/210927/cg-c001-eng.htm. Accessed 8 May 2022.

[40] Chirumbolo A, Callea A, Urbini F. The effect of job insecurity and life uncertainty on everyday consumptions and broader life projects during COVID-19 pandemic. Int J Environ Res Public Health. 2021;18. 10.3390/ijerph18105363 PMC8157599 34069918

[41] Wilson JM, Lee J, Fitzgerald HN, Oosterhoff B, Sevi B, Shook NJ (2020). Job insecurity and financial concern during the COVID-19 pandemic are associated with worse mental health. J Occup Environ Med.

[42] Fukushima N, Machida M, Kikuchi H, Amagasa S, Hayashi T, Odagiri Y (2021). Associations of working from home with occupational physical activity and sedentary behavior under the COVID-19 pandemic. J Occup Health.

[43] Ráthonyi G, Kósa K, Bács Z, Ráthonyi-Ódor K, Füzesi I, Lengyel P, et al. Changes in workers’ physical activity and sedentary behavior during the COVID-19 pandemic. Sustainability. 2021;13.

[44] Stockwell S, Trott M, Tully M, Shin J, Barnett Y, Butler L, et al. Changes in physical activity and sedentary behaviours from before to during the COVID-19 pandemic lockdown: A systematic review. BMJ Open Sport Exerc Med. 2021;7. 10.1136/bmjsem-2020-000960 PMC7852071 34192010

[45] Tremblay MS, Colley RC, Saunders TJ, Healy GN, Owen N (2010). Physiological and health implications of a sedentary lifestyle. Appl Physiol Nutr Metab.

[46] Smith L, Hamer M, Ucci M, Marmot A, Gardner B, Sawyer A, et al. Weekday and weekend patterns of objectively measured sitting, standing, and stepping in a sample of office-based workers: The active buildings study. BMC Public Health. 2015;15. 10.1186/s12889-014-1338-1 PMC4308830 25595219

[47] de Graaf R, van Dorsselaer S, ten Have M, Schoemaker C, Vollebergh WAM (2005). Seasonal variations in mental disorders in the general population of a country with a maritime climate: Findings from the Netherlands Mental Health Survey and Incidence Study. Am J Epidemiol.

[48] Reilly T, Peiser B (2006). Seasonal variations in health-related human physical activity. Sports Med.

[49] Faulkner J, O’Brien WJ, McGrane B, Wadsworth D, Batten J, Askew CD (2021). Physical activity, mental health and well-being of adults during initial COVID-19 containment strategies: A multi-country cross-sectional analysis. J Sci Med Sport.

[50] Faulkner J, O’Brien WJ, Stuart B, Stoner L, Batten J, Wadsworth D, et al. Physical activity, mental health and wellbeing of adults within and during the easing of COVID-19 restrictions, in the United Kingdom and New Zealand. Int J Environ Res Public Health. 2022;19. 10.3390/ijerph19031792 PMC8835233 35162815

[51] Hargreaves EA, Lee C, Jenkins M, Calverley JR, Hodge K, Houge Mackenzie S. Changes in physical activity pre-, during and post-lockdown COVID-19 restrictions in New Zealand and the explanatory role of daily hassles. Front Psychol. 2021;12. 10.3389/fpsyg.2021.642954 PMC7947353 33716912

[52] Lemay M. Understanding the mechanism of panel attrition. University of Maryland; 2009.

[53] Satherley N, Milojev P, Greaves LM, Huang Y, Osborne D, Bulbulia J (2015). Demographic and psychological predictors of panel attrition: Evidence from the New Zealand attitudes and values study. PLoS ONE.

