# Supplementary material for: Mental and physical health and well-being of canadian employees who were working from home during the COVID-19 pandemic
Source: BMC Public Health. 2022 Oct 31;22:1987. doi: 10.1186/s12889-022-14349-5 (PMC9619010; doi:10.1186/s12889-022-14349-5)
Supplement: Supplementary file 1 — Supplementary Material 1 [file 12889_2022_14349_MOESM1_ESM.docx]

# Initial survey

**National survey on the physical and mental health effects of working from home**

The goal of this survey is to understand the impact of working from home on employees’ health and well-being. Your responses may help inform the development of strategies to assist Canadian workplaces to better manage the current environment and to meet ongoing and future changes to the way we work. This survey is being conducted by the Canadian Institute for Safety, Wellness and Performance, and has been adapted and implemented in parallel with a nationwide Australian survey distributed by La Trobe University.

Completing the survey takes about 20 minutes. You can withdraw at any point by closing your web browser.

All participants who complete the survey have the opportunity to be entered into a draw to win a $50 gift card.

If you have questions, contact us at ahackney@conestogac.on.ca.

 Please click '-->' to read the Participant Information Statement and start the survey.

**Participant Information Statement**

- I consent to my responses being used in this research

Have you ever worked from home during the COVID19 pandemic?

- Yes
- No

When working at home during the COVID-19 pandemic, has this been at least 2 or more days every week?

- Yes
- No

What is your age group?

- Under 18 years
- 18 – 25 years
- 26 – 35 years
- 36 – 45 years
- 46 – 55 years
- 56 years and over
- Prefer not to say

Are you currently living in Canada?

- Yes
- No

While working at home during the COVID-19 pandemic, which province are/were you living in? (select from list)

- Alberta
- British Columbia
- Manitoba
- New Brunswick
- Newfoundland and Labrador
- Nova Scotia
- Ontario
- Prince Edward Island
- Quebec
- Saskatchewan
- Northwest Territories
- Nunavut
- Yukon
- Prefer not to say

Are you…

- Male
- Female
- Other
- Prefer not to say

Which industry do you mainly work in? (choose on from this list). NOTE: if you are a student with a part-time job, please complete the survey questions with respect to your part-time work.

- Accommodation and Food Services
- Agriculture, Forestry, Fishing (e.g., horticulture, nursery etc.)
- Arts, Recreation Services
- Construction
- Education and Training (e.g., teacher, college, and university professor)
- Electricity, Gas, Water and Waste Services
- Financial and Insurance Services
- Healthcare & Social Assistance
- Information, Media & Telecommunications
- Manufacturing
- Mining
- Other Services (e.g., hairdressing, beauty, tattooing, car mechanic etc.)
- Professional, Scientific, and Technical Services (e.g., research, engineer, legal, graduate student teaching/research assistant)
- Public Administration and Safety (e.g., police, ambulance, public servant etc.)
- Rental, Hiring & Real Estate Services
- Retail Trade
- Transport, Postal & Warehousing
- Wholesale Trade
- Other (please specify):

Please specify which *other* industry you mainly work in.

- Prefer not to say
- I am a full-time student with no part- or full-time work

Which sector are you employed in? (select one from the list)

- Public sector
- Private sector
- Not for profit sector
- Self-employed
- Prefer not to say

Which best describes your current main role (select one from the list). *NOTE: if you are a student working part time, please answer the survey questions with respect to your part time job.

- Manager (e.g., general managers, chief executives, other specialist managers)
- Professional (e.g., arts, academic, grad student, accountant, allied health, education, IT professional, scientist, engineer)
- Technician & Trade Workers (e.g., hairdresser, chef/cook, sound engineer, mechanic, plumber, electrician)
- Clerical or Administrative Workers (e.g., office worker, project administrator, bookkeeper, conveyancer)
- Community and Personal Service Worker (e.g., support workers, education aides, sports and fitness workers)
- Sales Workers (e.g., real estate agent, telemarketer, insurance)
- Machinery Operators & Drivers (e.g., truck driver, bus driver, controlling & monitoring machines)
- Labourers (e.g., professional cleaner, unloading freight, food preparation)
- I am a full-time student (e.g., no full time or part time job)
- Prefer not to say

What size business do you work in?

- Sole trader/self-employed
- 19 employees or less
- Between 20 and 199 employees
- More than 200 employees
- Prefer not to say

During the COVID-19 pandemic, which of the following best describes your usual living arrangements? You live:

- Alone
- With one or more adults (no children under 18 years)
- With one or more adults AND children aged 0 - 18 years
- With one or more children aged 0 - 18 years (no other adults)
- Prefer not to say

Throughout the COVID-19 pandemic, how many children (0-18 years) are/were you the primary caregiver for? (select one from the list)

- None
- 1
- 2
- 3
- 4
- 5
- More than 5
- Prefer not to say

When WORKING AT HOME during COVID-19, how many children are usually at home with you? Please answer this question in relation to your CURRENT situation.

- 1
- 2
- 3
- 4
- 5+
- Prefer not to say
- 0 (e.g., none of your children are at home with you during working hours)

What age group(s) are the children who are at home with you while you work?

- Less than 4 years
- 4-6 years
- 6-8 years
- 8-12 years
- 12-16 years
- 16-18 years
- Prefer not to say

Are you homeschooling your children?

- Yes
- No
- Prefer not to say

How many hours a day are you actively involved in your children education during COVID 19? (e.g., teaching and learning activities)

- 0
- 1-2 hours
- 2-3 hours
- 3-4 hours
- More than 4 hours
- Prefer not to say

How many days a week are your children attending classes virtually at home?

- 1 day
- 2 days
- 3 days
- 4 days
- 5 days
- Prefer not to say

Do you have primary care responsibilities (i.e., dependents) other than children?

- No
- Yes, adult(s) living with me
- Yes, adult(s) living elsewhere
- Other
- Prefer not to say

How satisfied are you with the way childcare tasks are divided between you and others in your household?

- Very dissatisfied
- Not satisfied
- Neutral
- Satisfied
- Very satisfied
- Prefer not to say

How satisfied are you with the way household tasks are divided between you and others in your household?

- Very dissatisfied
- Not satisfied
- Neutral
- Satisfied
- Very satisfied
- Not applicable
- Prefer not to say

Before the start of the COVID-19 pandemic (March 2020), how often per week did you work from home? (select one from the list)

- None/very occasionally
- 1 day
- 2 days
- 3 days
- 4 days
- 5 or more days
- Prefer not to say

Before the start of the COVID-19 pandemic, how long did you work from home? (select one from the list)

- 0 - 6 months before the pandemic
- 6 - 12 months before the pandemic
- More than 12 months before the pandemic
- Prefer not to say

When you are/were working at home during the COVID-19 pandemic, what are/were your usual working hours (average per week)?

- Full time (35 hours or more)
- 26 - 34 hrs
- 21 - 25 hrs
- 15 - 20 hrs
- 14 hrs or less
- Prefer not to say

When you are/were working at home during the pandemic, how many days per week do (did) you usually work from home? (select one from the list)

- 2 days
- 3 days
- 4 days
- 5 or more days
- Prefer not to say

From the start of the pandemic (March 2020) up to now, how many **MONTHS IN TOTAL** have you worked at home? (select one from the list)

- 1
- 2
- 3
- 4
- 5
- 6
- 7
- 8
- 9
- 10
- 11
- 12
- Prefer not to say

When you are working at home, where do you usually work?

- I just find a place somewhere that’s free, such as on the kitchen table or other place
- I have my own place in a separate room by myself
- I have my own place but in a room that can be busy with other people
- Prefer not to say

How comfortable is your home workstation (where you usually work at home) compared to your usual workstation before the COVID- 19 pandemic?

- Much less comfortable
- A bit less comfortable
- About the same
- A bit more comfortable
- Much more comfortable
- Prefer not to say

Do you use an adjustable chair?

- Yes
- No
- Prefer not to say

Please indicate which of the following technologies you use when working from home.

- Laptop Yes No
- Desktop Yes No
- Phone/Tablet Yes No
- Other devices Yes No

Please specify which *other* devices you use:

Do you use a separate keyboard and/or mouse with your laptop?

- Yes, both keyboard and mouse
- Yes, a mouse but not a keyboard
- Yes, a keyboard but not a mouse
- No
- Prefer not to say

Do you typically use a secondary screen with your desktop?

- Yes
- No
- Prefer not to say

Do you typically use a separate monitor with your laptop?

- Yes
- No
- Prefer not to say

When you began working from home during the pandemic, did your employer provide any suggestions to setting up your workstation?

- Yes
- No
- Prefer not to say

While working from home during the COVID-19 pandemic, did you use video conferencing to communicate with your employer or work colleagues?

- Yes
- No
- Prefer not to say

During the pandemic, how much time per day, on average, would you spend communicating with your employer or work colleague through video conferencing or telephone?

- Less than an hour
- Between 1 and 2 hours
- Between 2 and 3 hours
- More than 3 hours
- Prefer not to say

On a typical work-at-home day… During your work time, how much of the time would you usually spend:

|  | Percentage (in each row, enter a number 0-100, total should add up to 100%) |
| --- | --- |
| Sitting? |  |
| Standing? |  |
| Walking? |  |
| Performing heavy labour or physically demanding tasks? |  |
| Prefer not to say (enter ‘100’ into textbox) |  |

On that same day during your non-work time, how much of the time would you usually spend:

|  | Percentage (in each row, enter a number 0-100, total should add up to 100%) |
| --- | --- |
| Sitting? |  |
| Standing? |  |
| Walking? |  |
| Performing heavy labour or physically demanding tasks? |  |
| Prefer not to say (enter ‘100’ into textbox) |  |

While working at home during the COVID-19 pandemic…

| How often have you felt worn out? | Never | Seldom | Sometimes | Often | Always | Prefer not to say |
| --- | --- | --- | --- | --- | --- | --- |
| How often have you been physically exhausted? | Never | Seldom | Sometimes | Often | Always | Prefer not to say |
| How often have you been emotionally exhausted? | Never | Seldom | Sometimes | Often | Always | Prefer not to say |
| How often have you felt tired? | Never | Seldom | Sometimes | Often | Always | Prefer not to say |
| How often have you had problems relaxing? | Never | Seldom | Sometimes | Often | Always | Prefer not to say |
| How often have you been irritable? | Never | Seldom | Sometimes | Often | Always | Prefer not to say |
| How often have you been tense? | Never | Seldom | Sometimes | Often | Always | Prefer not to say |
| How often have you had stomach ache? | Never | Seldom | Sometimes | Often | Always | Prefer not to say |
| How often have you had a headache? | Never | Seldom | Sometimes | Often | Always | Prefer not to say |
| How often have you had problems concentrating? | Never | Seldom | Sometimes | Often | Always | Prefer not to say |
| How often have you found it difficult to think clearly? | Never | Seldom | Sometimes | Often | Always | Prefer not to say |
| How often have you had difficulty making decisions? | Never | Seldom | Sometimes | Often | Always | Prefer not to say |
| How often have you had difficulty remembering things? | Never | Seldom | Sometimes | Often | Always | Prefer not to say |

In general, would you say your physical health is…

- Poor
- Fair
- Good
- Very Good
- Excellent
- Prefer not to say

In general, would you say your mental health is…

- Poor
- Fair
- Good
- Very Good
- Excellent
- Prefer not to say

Thinking about the balance between your work and home life when you are working at home during the COVID-19 pandemic, to what extent do you agree with the following statements?

| The demands of my work interfere with my home and family life | Strongly disagree | Disagree | Slightly disagree | Neither agree nor disagree | Slightly agree | Agree | Strongly agree | Prefer not to say |
| --- | --- | --- | --- | --- | --- | --- | --- | --- |
| The amount of time my job takes up makes it difficult to fulfill family responsibilities | Strongly disagree | Disagree | Slightly disagree | Neither agree nor disagree | Slightly agree | Agree | Strongly agree | Prefer not to say |
| Things I want to do at home do not get done because of the demands my job puts on me | Strongly disagree | Disagree | Slightly disagree | Neither agree nor disagree | Slightly agree | Agree | Strongly agree | Prefer not to say |
| My job creates stresses that make it difficult for me to fulfil family duties | Strongly disagree | Disagree | Slightly disagree | Neither agree nor disagree | Slightly agree | Agree | Strongly agree | Prefer not to say |
| Due to work-related duties, I have to make changes to my plans for family activities | Strongly disagree | Disagree | Slightly disagree | Neither agree nor disagree | Slightly agree | Agree | Strongly agree | Prefer not to say |
| The demands of my family or spouse/partner interfere with work-related activities | Strongly disagree | Disagree | Slightly disagree | Neither agree nor disagree | Slightly agree | Agree | Strongly agree | Prefer not to say |
| I have to put off doing things at work because of demands on my time at home | Strongly disagree | Disagree | Slightly disagree | Neither agree nor disagree | Slightly agree | Agree | Strongly agree | Prefer not to say |
| Things I want to do for work don't get done because of the demands of my family or spouse/partner | Strongly disagree | Disagree | Slightly disagree | Neither agree nor disagree | Slightly agree | Agree | Strongly agree | Prefer not to say |
| My home life interferes with my work responsibilities such as starting work on time, accomplishing daily tasks and working overtime | Strongly disagree | Disagree | Slightly disagree | Neither agree nor disagree | Slightly agree | Agree | Strongly agree | Prefer not to say |
| Family-related stress interferes with my ability to perform job-related duties | Strongly disagree | Disagree | Slightly disagree | Neither agree nor disagree | Slightly agree | Agree | Strongly agree | Prefer not to say |

The following questions are about your experience of working at home during the COVID-19 pandemic.

| I do not have time to complete all my work tasks | Never | Seldom | Sometimes | Often | Always | Prefer not to say |
| --- | --- | --- | --- | --- | --- | --- |
| I get behind in my work | Never | Seldom | Sometimes | Often | Always | Prefer not to say |
| My work is unevenly distributed so it piles up | Never | Seldom | Sometimes | Often | Always | Prefer not to say |
| I have enough time for my work tasks | Never | Seldom | Sometimes | Often | Always | Prefer not to say |
| I have a large degree of influence on decisions affecting my work | Never | Seldom | Sometimes | Often | Always | Prefer not to say |
| I can influence the amount of work assigned to me | Never | Seldom | Sometimes | Often | Always | Prefer not to say |
| I have some influence on the particular work tasks I have to do | Never | Seldom | Sometimes | Often | Always | Prefer not to say |

The following questions are about your experience of working at home during the COVID-19 pandemic.

| There is a good atmosphere between me and my colleagues | Never | Seldom | Sometimes | Often | Always | Prefer not to say |
| --- | --- | --- | --- | --- | --- | --- |
| I feel part of a work community | Never | Seldom | Sometimes | Often | Always | Prefer not to say |
| I can get help and support from my immediate supervisor, if needed | Never | Seldom | Sometimes | Often | Always | Prefer not to say |
| My immediate supervisor is willing to listen to my problems, if needed | Never | Seldom | Sometimes | Often | Always | Prefer not to say |
| I can get help and support from my work colleagues, if needed | Never | Seldom | Sometimes | Often | Always | Prefer not to say |
| My colleagues are willing to listen to my problems, if needed | Never | Seldom | Sometimes | Often | Always | Prefer not to say |

The following questions are about your experience of working at home during the COVID-19 pandemic.

| I am informed well in advance about important decisions, changes, or plans for the future | To a very small extent | To a small extent | Somewhat | To a large extent | To a very large extent | Prefer not to say |
| --- | --- | --- | --- | --- | --- | --- |
| I receive all the information I need to do my work well | To a very small extent | To a small extent | Somewhat | To a large extent | To a very large extent | Prefer not to say |
| My work has clear objectives | To a very small extent | To a small extent | Somewhat | To a large extent | To a very large extent | Prefer not to say |
| I know exactly which things are my responsibility | To a very small extent | To a small extent | Somewhat | To a large extent | To a very large extent | Prefer not to say |
| I know exactly what is expected of my | To a very small extent | To a small extent | Somewhat | To a large extent | To a very large extent | Prefer not to say |
| Contradictory work demands are placed on me | To a very small extent | To a small extent | Somewhat | To a large extent | To a very large extent | Prefer not to say |
| I sometimes have to do things that ought to have been done in a different way | To a very small extent | To a small extent | Somewhat | To a large extent | To a very large extent | Prefer not to say |

The following questions are about your experience of working at home during the COVID-19 pandemic.

| My supervisor is good at work planning | To a very small extent | To a small extent | Somewhat | To a large extent | To a very large extent | Not applicable | Prefer not to say |
| --- | --- | --- | --- | --- | --- | --- | --- |
| My supervisor is good at solving conflicts | To a very small extent | To a small extent | Somewhat | To a large extent | To a very large extent | Not applicable | Prefer not to say |
| My work is recognized and appreciated by supervisors and managers | To a very small extent | To a small extent | Somewhat | To a large extent | To a very large extent | Not applicable | Prefer not to say |
| I am treated fairly by my supervisors or managers | To a very small extent | To a small extent | Somewhat | To a large extent | To a very large extent | Not applicable | Prefer not to say |

The following questions are about your experience of working at home during the COVID-19 pandemic.

| Conflicts are resolved in a fair way | To a very small extent | To a small extent | Somewhat | To a large extent | To a very large extent | Not applicable | Prefer not to say |
| --- | --- | --- | --- | --- | --- | --- | --- |
| Work is distributed fairly | To a very small extent | To a small extent | Somewhat | To a large extent | To a very large extent | Not applicable | Prefer not to say |
| I am worried about becoming unemployed | To a very small extent | To a small extent | Somewhat | To a large extent | To a very large extent | Not applicable | Prefer not to say |
| I am worried about difficulty finding another job if I become unemployed | To a very small extent | To a small extent | Somewhat | To a large extent | To a very large extent | Not applicable | Prefer not to say |
| I am worried about being transferred to another job against my will | To a very small extent | To a small extent | Somewhat | To a large extent | To a very large extent | Not applicable | Prefer not to say |
| I am worried about a possible decrease in salary | To a very small extent | To a small extent | Somewhat | To a large extent | To a very large extent | Not applicable | Prefer not to say |
| Management trusts employees to do their work well | To a very small extent | To a small extent | Somewhat | To a large extent | To a very large extent | Not applicable | Prefer not to say |
| Management shares enough information with me | To a very small extent | To a small extent | Somewhat | To a large extent | To a very large extent | Not applicable | Prefer not to say |
| Employees can trust information that comes from management | To a very small extent | To a small extent | Somewhat | To a large extent | To a very large extent | Not applicable | Prefer not to say |

How pleased are you with your job overall, everything taken into consideration?

- Very unsatisfied
- Unsatisfied
- Neither unsatisfied or satisfied
- Satisfied
- Very satisfied
- Prefer not to say

For the next set of questions, **COMPARE** how things are/were while working **AT HOME** during the COVID-19 pandemic, with your work experience prior to the pandemic (March 2020). When working at home during the COVID-19 pandemic...

| I can get help and feedback from my work colleagues, if needed | Much less than before | Somewhat less than before | Same as before | Somewhat more than before | Much more than before | Prefer not to say |
| --- | --- | --- | --- | --- | --- | --- |
| I can get help and feedback from my immediate supervisor, if needed | Much less than before | Somewhat less than before | Same as before | Somewhat more than before | Much more than before | Prefer not to say |
| I receive information that keeps me in touch with workplace events and developments | Much less than before | Somewhat less than before | Same as before | Somewhat more than before | Much more than before | Prefer not to say |
| I feel a good sense of community with my work colleagues | Much less than before | Somewhat less than before | Same as before | Somewhat more than before | Much more than before | Prefer not to say |
| Trying to work productively is stressful or frustrating | Much less than before | Somewhat less than before | Same as before | Somewhat more than before | Much more than before | Prefer not to say |
| Work interferes with my home or family life | Much less than before | Somewhat less than before | Same as before | Somewhat more than before | Much more than before | Prefer not to say |
| I often feel tired or exhausted | Much less than before | Somewhat less than before | Same as before | Somewhat more than before | Much more than before | Prefer not to say |
| I enjoy my work and the job overall | Much less than before | Somewhat less than before | Same as before | Somewhat more than before | Much more than before | Prefer not to say |

In the last 6 months, have you ever experienced discomfort or pain in any part of your body, especially towards the end of your working day or night?

- Yes
- No
- Prefer not to say

In the last 6 months... **HOW OFTEN** have you felt discomfort or pain in your **NECK AND SHOULDERS**?

- Never
- Occasionally
- Sometimes
- Often
- Almost always
- Prefer not to say

In the last 6 months... **HOW BAD** was the discomfort or pain in your **NECK AND SHOULDERS?**

- Mild
- Moderate
- Severe
- Prefer not to say

In the last 6 months... **HOW OFTEN** have you felt discomfort or pain in your **HANDS AND FINGERS**?

- Never
- Occasionally
- Sometimes
- Often
- Almost always
- Prefer not to say

In the last 6 months... **HOW BAD** was the discomfort or pain in your **HANDS AND FINGERS**?

- Mild
- Moderate
- Severe
- Prefer not to say

In the last 6 months... **HOW OFTEN**have you felt discomfort or pain in your **ARMS**?

- Never
- Occasionally
- Sometimes
- Often
- Almost always
- Prefer not to say

In the last 6 months... **HOW BAD** was the discomfort or pain in your **ARMS**?

- Mild
- Moderate
- Severe
- Prefer not to say

In the last 6 months... **HOW OFTEN** have you felt discomfort or pain in your **MIDDLE AND/OR LOWER BACK**?

- Never
- Occasionally
- Sometimes
- Often
- Almost always
- Prefer not to say

In the last 6 months... **HOW BAD** was the discomfort or pain in your **MIDDLE AND/OR LOWER BACK**?

- Mild
- Moderate
- Severe
- Prefer not to say

In the last 6 months... **HOW OFTEN** have you felt discomfort or pain in your **HIPS, BOTTOM, LEGS OR FEET**?

- Never
- Occasionally
- Sometimes
- Often
- Almost always
- Prefer not to say

In the last 6 months... **HOW BAD** was the discomfort or pain in your **HIPS, BOTTOM, LEGS OR FEET**?

- Mild
- Moderate
- Severe
- Prefer not to say

Which of the following hardware has your **EMPLOYER** provided for you to use at home (tick all that apply)?

- None
- Desktop computer
- Laptop computer
- Separate keyboard for laptop
- Mouse
- Smart Phone
- Tablet
- Other

Please specify which *other* hardware is provided:

- Prefer not to say

Which of the following software has your **EMPLOYER** provided for you to use at home (tick all that apply)?

- None
- Online meeting platform (e.g. Zoom, Webex, Microsoft Teams)
- Software program (e.g. Word, Excel)
- Access to organizational network (e.g. shared drives, intranet)
- Other
- Prefer not to say

Which of the following hardware have you provided **YOURSELF**, for use at home (tick all that apply)?

- None
- Desktop computer
- Laptop computer
- Separate keyboard for laptop
- Mouse
- Smart Phone
- Tablet
- Other

Please specify which *other* hardware you have provided for yourself:

- Prefer not to say

Which of the following software has your **YOURSELF** provided for you to use at home (tick all that apply)?

- None
- Online meeting platform (e.g. Zoom, Webex, Microsoft Teams)
- Software program (e.g. Word, Excel)
- Access to organization network (e.g. shared drives, intranet)
- Other
- Prefer not to say

Technology support

| I have good quality internet connection at home | Strongly disagree | Disagree | Neither agree nor disagree | Agree | Strongly Agree | Not Applicable | Prefer not to say |
| --- | --- | --- | --- | --- | --- | --- | --- |
| I can get good help and support from work if I have technology (hardware or software) problems | Strongly disagree | Disagree | Neither agree nor disagree | Agree | Strongly Agree | Not Applicable | Prefer not to say |
| I can get good support from family or friends to help me if I have technology (hardware or software) problems | Strongly disagree | Disagree | Neither agree nor disagree | Agree | Strongly Agree | Not Applicable | Prefer not to say |

Productivity

| The technical hardware I use when working at home (e.g., laptop, phone) enables me to work effectively | Strongly disagree | Disagree | Neither agree nor disagree | Agree | Strongly Agree | Not Applicable | Prefer not to say |
| --- | --- | --- | --- | --- | --- | --- | --- |
| The software I use when working at home enables me to work effectively | Strongly disagree | Disagree | Neither agree nor disagree | Agree | Strongly Agree | Not Applicable | Prefer not to say |

Technology complexity

| I could work more effectively at home if I new more about technology | Strongly disagree | Disagree | Neither agree nor disagree | Agree | Strongly Agree | Not Applicable | Prefer not to say |
| --- | --- | --- | --- | --- | --- | --- | --- |
| I find it difficult to learn how to use new technologies | Strongly disagree | Disagree | Neither agree nor disagree | Agree | Strongly Agree | Not Applicable | Prefer not to say |

Taking everything into account, how many DAYS PER WEEK would you PREFER to work at home during a NON-PANDEMIC TIME?

- None
- 1
- 2
- 3
- 4
- Every day
- Prefer not to say

Taking everything into account, how many DAYS PER WEEK would you PREFER to work at home when your perceived risk of COVID-19 infection is LOW?

- None
- 1
- 2
- 3
- 4
- Every day
- Prefer not to say

Taking everything into account, how many DAYS PER WEEK would you PREFER to work from home when your perceived risk of COVID-19 infection is HIGH?

- None
- 1
- 2
- 3
- 4
- Every day
- Prefer not to say

You are now at the end of the survey. Thank you very much for your time. Before you go… we have some further opportunities that we invite you to consider:

Would you like to be entered into a draw to win a $50 gift card?

- Yes
- No

To find how well people are coping longer term, we would like to survey you again in 6-months’ time. Do you agree to be contacted for this additional survey?

- Yes
- No
