# Supplementary material for: Mental and physical health and well-being of canadian employees who were working from home during the COVID-19 pandemic
Source: BMC Public Health. 2022 Oct 31;22:1987. doi: 10.1186/s12889-022-14349-5 (PMC9619010; doi:10.1186/s12889-022-14349-5)
Supplement: Supplementary file 2 — Supplementary Material 2 [file 12889_2022_14349_MOESM2_ESM.docx]

# Follow-up survey

**National survey on the physical and mental health effects of working from home**

You are invited to participate in this research study because you completed the initial work-from-home survey conducted by the Canadian Institute for Safety, Wellness & Performance (CISWP) and indicated that you would like to be contacted for participation in the follow-up survey. The goal of these surveys is to understand the impact of working from home on employees’ health and well-being and whether these impacts change over time. Your responses may help inform the development of strategies to assist Canadian workplaces to better manage the current environment and to meet ongoing and future changes to the way we work. This survey has been adapted and implemented in parallel with a nationwide Australian survey distributed by La Trobe University.

Completing the survey takes about 20 minutes. You can withdraw at any point by closing your web browser.

All participants who complete the survey can choose to be entered into a draw to win a $50 gift card.

If you have questions, contact us at ahackney@conestogac.on.ca.

 Please click '-->' to read the Participant Information Statement and start the survey.

**Participant Information Statement**

- I consent to my responses being used in this research

What is your age group?

- Under 18 years
- 18 – 25 years
- 26 – 35 years
- 36 – 45 years
- 46 – 55 years
- 56 years and over
- Prefer not to say

What is your gender?

- Man
- Woman
- Non-binary
- Prefer not to disclose
- Prefer to self-disclose

*What is your self-disclosed gender?*

Are you currently living in Canada?

- Yes
- No

What province/territory do you currently reside? (select from list)

- Alberta
- British Columbia
- Manitoba
- New Brunswick
- Newfoundland and Labrador
- Nova Scotia
- Ontario
- Prince Edward Island
- Quebec
- Saskatchewan
- Northwest Territories
- Nunavut
- Yukon
- Prefer not to say

In the last 6 months, have you become unemployed?

- Yes
- No

In the last 6 months, have you started a new job?

- Yes
- No

In the last 6 months, have you had a change in pay/salary? (choose all that apply)

- Yes, it has increased
- Yes, it has decreased
- No, it has stayed the same
- Prefer not to say

Which industry do you mainly work in? (choose one from this list).

- Accommodation and Food Services
- Agriculture, Forestry, Fishing (e.g., horticulture, nursery etc.)
- Arts and Recreation Services
- Construction
- Education and Training (e.g., teacher, college/university professor)
- Electricity, Gas, Water and Waste Services
- Financial and Insurance Services
- Healthcare and Social Assistance
- Information, Media and Telecommunications
- Manufacturing
- Mining
- Other Services (i.e., hairdressing, beauty, tattooing, mechanic etc.)
- Professional, Scientific, and Technical Services
- Public Administration
- Rental, Hiring and Real Estate Services
- Retail
- Transport, Postal and Warehousing
- Wholesale
- Other

*Please specify which other industry you mainly work in.*

- Prefer not to say

Which sector are you employed in? (select one from the list)

- Public sector
- Private sector
- Not for profit sector
- Self employed
- Prefer not to say

Which best describes your main role (select one from the list).

- Manager (e.g., general managers, chief executives, other specialist managers)
- Professional (e.g., arts, academic, grad student, accountant, allied health, education, IT professional, scientist, engineer)
- Technician & Trade Workers (e.g., hairdresser, chef/cook, sound engineer, mechanic, plumber, electrician)
- Clerical or Administrative Workers (e.g., office worker, project administrator, bookkeeper, conveyancer)
- Community and Personal Service Worker (e.g., support workers, education aides, sports and fitness workers)
- Sales Worker (e.g., real estate agent, telemarketer, insurance)
- Machinery Operators & Drivers (e.g., truck driver, bus driver, controlling & monitoring machines)
- Labourers (e.g., professional cleaner, unloading freight, food preparation)
- Prefer not to say

What size business do you work in?

- Sole trader/self-employed
- 19 employees or less
- Between 20 and 199 employees
- More than 200 employees
- Prefer not to say

Compared to prior to the pandemic (March 11, 2020), the number of **vacation and sick days** that you typically use has…

- Increased
- Decreased
- Stayed the same
- Prefer not to say

Are you satisfied by the amount of **sick days**, and flexibility that is available to you from your employer?

- Yes
- No
- Prefer not to say

In the last 6 months, which statement best describes your working arrangement?

- I continued to work from home the entire time
- I returned to the workplace on a flexible arrangement (i.e., I split my time between working outside the home and working from home)
- I returned to the workplace 100% of the time, but only for a short period, and then returned to working from home 100% of the time
- Other

*Please* *describe your working arrangement.*

In the last 6 months, which of the following best describes your usual living arrangements? You live:

- Alone
- With one or more adults (no children under 18 years)
- With one or more adults AND children aged 0 - 18 years
- With one or more children aged 0 - 18 years (no other adults)
- Prefer not to say

Within the last 6 months, how many children (0-18 years) were you the primary caregiver for? (select one from the list)

- None
- 1
- 2
- 3
- 4
- 5
- More than 5
- Prefer not to say

When **working from home** within the last 6 months, how many children are usually at home with you?

- 1
- 2
- 3
- 4
- 5+
- Prefer not to say
- 0 (none of your children are at home with you during working hours)

What age group(s) are the children who are at home with you while you work? (select all that apply)

- Less than 4 years
- 4-6 years
- 6-8 years
- 8-12 years
- 12-16 years
- 16-18 years
- Prefer not to say

Are you homeschooling your children?

- Yes
- No
- Prefer not to say

In the last 6 months, how many hours a day are you actively involved in your children’s education? (e.g., teaching and learning activities)

- 0
- 1-2 hours
- 2-3 hours
- 3-4 hours
- More than 4 hours
- Prefer not to say

In the last 6 months, on average, how many days a week are your children attending classes virtually at home?

- 1 day
- 2 days
- 3 days
- 4 days
- 5 days
- Prefer not to say

In the last 6 months, did you have primary care responsibilities (i.e., dependents) other than children?

- No
- Yes, adult(s) living with me
- Yes, adult(s) living elsewhere
- Other

*Please describe what* other *primary care responsibilities you have.*

- Prefer not to say

How satisfied are you with the way **childcare** tasks are divided between you and others in your household?

- Very dissatisfied
- Not satisfied
- Neutral
- Satisfied
- Very satisfied
- Prefer not to say

How satisfied are you with the way **household** tasks are divided between you and others in your household?

- Very dissatisfied
- Not satisfied
- Neutral
- Satisfied
- Very satisfied
- Not applicable
- Prefer not to say

When you are working at home, where do you usually work?

- I just find a place somewhere that’s free, such as on the kitchen table or other place
- I have my own place in a separate room by myself
- I have my own place but in a room that can be busy with other people
- Prefer not to say

How comfortable is your home workstation (where you usually work at home) compared to your usual workstation before the COVID- 19 pandemic (March 11, 2020)?

- Much less comfortable
- A bit less comfortable
- About the same
- A bit more comfortable
- Much more comfortable
- Prefer not to say

Do you use an adjustable chair?

- Yes
- No
- Prefer not to say

Please indicate which of the following technologies you use when working from home

- Laptop Yes No
- Desktop Yes No
- Phone/Tablet Yes No
- Other devices Yes No

*Please specify which* other *devices you use.*

Do you use a separate keyboard and/or mouse with your laptop?

- Yes, both keyboard and mouse
- Yes, a mouse but not a keyboard
- Yes, a keyboard but not a mouse
- No
- Prefer not to say

Do you typically use a secondary screen with your desktop?

- Yes
- No
- Prefer not to say

Do you typically use a separate monitor with your laptop?

- Yes
- No
- Prefer not to say

Have you received any additional training or suggestions (e.g. office ergonomic training) about your workstation setup from your employer in the past 6 months.?

- Yes
- No
- I do not remember
- Prefer not to say

While **working from home** during the last **6 months,** how often have you:

| Felt worn out? | Never | Seldom | Sometimes | Often | Always | Prefer not to say |
| --- | --- | --- | --- | --- | --- | --- |
| Been physically exhausted? | Never | Seldom | Sometimes | Often | Always | Prefer not to say |
| Been emotionally exhausted? | Never | Seldom | Sometimes | Often | Always | Prefer not to say |
| Felt tired? | Never | Seldom | Sometimes | Often | Always | Prefer not to say |
| Had problems relaxing? | Never | Seldom | Sometimes | Often | Always | Prefer not to say |
| Been irritable? | Never | Seldom | Sometimes | Often | Always | Prefer not to say |
| Been tense? | Never | Seldom | Sometimes | Often | Always | Prefer not to say |
| Had stomach ache? | Never | Seldom | Sometimes | Often | Always | Prefer not to say |
| Had a headache? | Never | Seldom | Sometimes | Often | Always | Prefer not to say |
| Had problems concentrating? | Never | Seldom | Sometimes | Often | Always | Prefer not to say |
| Found it difficult to think clearly? | Never | Seldom | Sometimes | Often | Always | Prefer not to say |
| Had difficulty making decisions? | Never | Seldom | Sometimes | Often | Always | Prefer not to say |
| Had difficulty remembering things? | Never | Seldom | Sometimes | Often | Always | Prefer not to say |

In general, would you say your **physical health** is…

- Poor
- Fair
- Good
- Very Good
- Excellent
- Prefer not to say

In general, would you say your **mental health** is…

- Poor
- Fair
- Good
- Very Good
- Excellent
- Prefer not to say

Thinking about the **balance between your work and home life** while working from home over the last 6 months, to what extent do you agree with the following statements?

| The demands of my work interfere with my home and family life | Strongly disagree | Disagree | Slightly disagreed | Neither agree nor disagreed | Slightly agree | Agree | Strongly agree | Prefer not to say |
| --- | --- | --- | --- | --- | --- | --- | --- | --- |
| The amount of time my job takes up makes it difficult to fulfill family responsibilities | Strongly disagree | Disagree | Slightly disagreed | Neither agree nor disagreed | Slightly agree | Agree | Strongly agree | Prefer not to say |
| Things I want to do at home do not get done because of the demands my job puts on me | Strongly disagree | Disagree | Slightly disagreed | Neither agree nor disagreed | Slightly agree | Agree | Strongly agree | Prefer not to say |
| My job creates stresses that make it difficult for me to fulfil family duties | Strongly disagree | Disagree | Slightly disagreed | Neither agree nor disagreed | Slightly agree | Agree | Strongly agree | Prefer not to say |
| Due to work-related duties, I have to make changes to my plans for family activities | Strongly disagree | Disagree | Slightly disagreed | Neither agree nor disagreed | Slightly agree | Agree | Strongly agree | Prefer not to say |
| The demands of my family or spouse/partner interfere with work-related activities | Strongly disagree | Disagree | Slightly disagreed | Neither agree nor disagreed | Slightly agree | Agree | Strongly agree | Prefer not to say |
| I have to put off doing things at work because of demands on my time at home | Strongly disagree | Disagree | Slightly disagreed | Neither agree nor disagreed | Slightly agree | Agree | Strongly agree | Prefer not to say |
| Things I want to do for work don't get done because of the demands of my family or spouse/partner | Strongly disagree | Disagree | Slightly disagreed | Neither agree nor disagreed | Slightly agree | Agree | Strongly agree | Prefer not to say |
| My home life interferes with my work responsibilities such as starting work on time, accomplishing daily tasks and working overtime | Strongly disagree | Disagree | Slightly disagreed | Neither agree nor disagreed | Slightly agree | Agree | Strongly agree | Prefer not to say |
| Family-related stress interferes with my ability to perform job-related duties | Strongly disagree | Disagree | Slightly disagreed | Neither agree nor disagreed | Slightly agree | Agree | Strongly agree | Prefer not to say |

The following questions are about your **experience of working from home** over the last 6 months.

| I do not have time to complete all my work tasks | Never | Seldom | Sometimes | Often | Always | Prefer not to say |
| --- | --- | --- | --- | --- | --- | --- |
| I get behind in my work | Never | Seldom | Sometimes | Often | Always | Prefer not to say |
| My work is unevenly distributed so it piles up | Never | Seldom | Sometimes | Often | Always | Prefer not to say |
| I have enough time for my work tasks | Never | Seldom | Sometimes | Often | Always | Prefer not to say |
| I have a large degree of influence on decisions affecting my work | Never | Seldom | Sometimes | Often | Always | Prefer not to say |
| I can influence the amount of work assigned to me | Never | Seldom | Sometimes | Often | Always | Prefer not to say |
| I have some influence on the particular work tasks I have to do | Never | Seldom | Sometimes | Often | Always | Prefer not to say |

The following questions are about your **experience of working from home** during the last 6 months.

| There is a good atmosphere between me and my colleagues | Never | Seldom | Sometimes | Often | Always | Prefer not to say |
| --- | --- | --- | --- | --- | --- | --- |
| I feel part of a work community | Never | Seldom | Sometimes | Often | Always | Prefer not to say |
| I can get help and support from my immediate supervisor, if needed | Never | Seldom | Sometimes | Often | Always | Prefer not to say |
| My immediate supervisor is willing to listen to my problems, if needed | Never | Seldom | Sometimes | Often | Always | Prefer not to say |
| I can get help and support from my work colleagues, if needed | Never | Seldom | Sometimes | Often | Always | Prefer not to say |
| My colleagues are willing to listen to my problems, if needed | Never | Seldom | Sometimes | Often | Always | Prefer not to say |

The following questions are about your **experience of working from home** in the last 6 months.

| I am informed well in advance about important decisions, changes, or plans for the future | To a very small extent | To a small extent | Somewhat | To a large extent | To a very large extent | Prefer not to say |
| --- | --- | --- | --- | --- | --- | --- |
| I receive all the information I need to do my work well | To a very small extent | To a small extent | Somewhat | To a large extent | To a very large extent | Prefer not to say |
| My work has clear objectives | To a very small extent | To a small extent | Somewhat | To a large extent | To a very large extent | Prefer not to say |
| I know exactly which things are my responsibility | To a very small extent | To a small extent | Somewhat | To a large extent | To a very large extent | Prefer not to say |
| I know exactly what is expected of my | To a very small extent | To a small extent | Somewhat | To a large extent | To a very large extent | Prefer not to say |
| Contradictory work demands are placed on me | To a very small extent | To a small extent | Somewhat | To a large extent | To a very large extent | Prefer not to say |
| I sometimes have to do things that ought to have been done in a different way | To a very small extent | To a small extent | Somewhat | To a large extent | To a very large extent | Prefer not to say |

The following questions are about your **experience of working from home** in the last 6 months.

| My supervisor is good at work planning | To a very small extent | To a small extent | Somewhat | To a large extent | To a very large extent | Not applicable | Prefer not to say |
| --- | --- | --- | --- | --- | --- | --- | --- |
| My supervisor is good at solving conflicts | To a very small extent | To a small extent | Somewhat | To a large extent | To a very large extent | Not applicable | Prefer not to say |
| My work is recognized and appreciated by supervisors and managers | To a very small extent | To a small extent | Somewhat | To a large extent | To a very large extent | Not applicable | Prefer not to say |
| I am treated fairly by my supervisors or managers | To a very small extent | To a small extent | Somewhat | To a large extent | To a very large extent | Not applicable | Prefer not to say |

The following questions are about your **experience of working from home** in the last 6 months.

| Conflicts are resolved in a fair way | To a very small extent | To a small extent | Somewhat | To a large extent | To a very large extent | Not applicable | Prefer not to say |
| --- | --- | --- | --- | --- | --- | --- | --- |
| Work is distributed fairly | To a very small extent | To a small extent | Somewhat | To a large extent | To a very large extent | Not applicable | Prefer not to say |
| I am worried about becoming unemployed | To a very small extent | To a small extent | Somewhat | To a large extent | To a very large extent | Not applicable | Prefer not to say |
| I am worried about difficulty finding another job if I become unemployed | To a very small extent | To a small extent | Somewhat | To a large extent | To a very large extent | Not applicable | Prefer not to say |
| I am worried about being transferred to another job against my will | To a very small extent | To a small extent | Somewhat | To a large extent | To a very large extent | Not applicable | Prefer not to say |
| I am worried about a possible decrease in salary | To a very small extent | To a small extent | Somewhat | To a large extent | To a very large extent | Not applicable | Prefer not to say |
| Management trusts employees to do their work well | To a very small extent | To a small extent | Somewhat | To a large extent | To a very large extent | Not applicable | Prefer not to say |
| Management shares enough information with me | To a very small extent | To a small extent | Somewhat | To a large extent | To a very large extent | Not applicable | Prefer not to say |
| Employees can trust information that comes from management | To a very small extent | To a small extent | Somewhat | To a large extent | To a very large extent | Not applicable | Prefer not to say |

How pleased are you with your job overall, everything taken into consideration?

- Very unsatisfied
- Unsatisfied
- Neither unsatisfied or satisfied
- Satisfied
- Very satisfied
- Prefer not to say

For the next set of questions, **COMPARE** how things are **NOW** while working from home to **6 months ago.**

| I can get help and feedback from my work colleagues, if needed | Much less than before | Somewhat less than before | Same as before | Somewhat more than before | Much more than before | Prefer not to say |
| --- | --- | --- | --- | --- | --- | --- |
| I can get help and feedback from my immediate supervisor, if needed | Much less than before | Somewhat less than before | Same as before | Somewhat more than before | Much more than before | Prefer not to say |
| I receive information that keeps me in touch with workplace events and developments | Much less than before | Somewhat less than before | Same as before | Somewhat more than before | Much more than before | Prefer not to say |
| I feel a good sense of community with my work colleagues | Much less than before | Somewhat less than before | Same as before | Somewhat more than before | Much more than before | Prefer not to say |
| Trying to work productively is stressful or frustrating | Much less than before | Somewhat less than before | Same as before | Somewhat more than before | Much more than before | Prefer not to say |
| Work interferes with my home or family life | Much less than before | Somewhat less than before | Same as before | Somewhat more than before | Much more than before | Prefer not to say |
| I often feel tired or exhausted | Much less than before | Somewhat less than before | Same as before | Somewhat more than before | Much more than before | Prefer not to say |
| I enjoy my work and the job overall | Much less than before | Somewhat less than before | Same as before | Somewhat more than before | Much more than before | Prefer not to say |

In the last 6 months, have you ever experienced discomfort or pain in any part of your body, especially towards the end of your working day or night?

- Yes
- No
- Prefer not to say

In the last 6 months... **HOW OFTEN** have you felt discomfort or pain in your **NECK AND SHOULDERS**?

- Never
- Occasionally
- Sometimes
- Often
- Almost always
- Prefer not to say

In the last 6 months... **HOW BAD** was the discomfort or pain in your **NECK AND SHOULDERS?**

- Mild
- Moderate
- Severe
- Prefer not to say

In the last 6 months... **HOW OFTEN** have you felt discomfort or pain in your **HANDS AND FINGERS**?

- Never
- Occasionally
- Sometimes
- Often
- Almost always
- Prefer not to say

In the last 6 months... **HOW BAD** was the discomfort or pain in your **HANDS AND FINGERS**?

- Mild
- Moderate
- Severe
- Prefer not to say

In the last 6 months... **HOW OFTEN**have you felt discomfort or pain in your **ARMS**?

- Never
- Occasionally
- Sometimes
- Often
- Almost always
- Prefer not to say

In the last 6 months... **HOW BAD** was the discomfort or pain in your **ARMS**?

- Mild
- Moderate
- Severe
- Prefer not to say

In the last 6 months... **HOW OFTEN** have you felt discomfort or pain in your **MIDDLE AND/OR LOWER BACK**?

- Never
- Occasionally
- Sometimes
- Often
- Almost always
- Prefer not to say

In the last 6 months... **HOW BAD** was the discomfort or pain in your **MIDDLE AND/OR LOWER BACK**?

- Mild
- Moderate
- Severe
- Prefer not to say

In the last 6 months... **HOW OFTEN** have you felt discomfort or pain in your **HIPS, BOTTOM, LEGS OR FEET**?

- Never
- Occasionally
- Sometimes
- Often
- Almost always
- Prefer not to say

In the last 6 months... **HOW BAD** was the discomfort or pain in your **HIPS, BOTTOM, LEGS OR FEET**?

- Mild
- Moderate
- Severe
- Prefer not to say

Indicate your level of agreement with the following statements about your productivity while working from home.

| The technical hardware I use when working from home (e.g., laptop, phone) enables me to work effectively | Strongly disagree | Disagree | Neither agree nor disagree | Agree | Strongly Agree | Not Applicable | Prefer not to say |
| --- | --- | --- | --- | --- | --- | --- | --- |
| The software I use when working at home enables me to work effectively | Strongly disagree | Disagree | Neither agree nor disagree | Agree | Strongly Agree | Not Applicable | Prefer not to say |
| I leave work tasks incomplete. | Strongly disagree | Disagree | Neither agree nor disagree | Agree | Strongly Agree | Not Applicable | Prefer not to say |
| I experience more disruptions while working from home than I do in my office at work. | Strongly disagree | Disagree | Neither agree nor disagree | Agree | Strongly Agree | Not Applicable | Prefer not to say |
| Working from home allows me to be more productive | Strongly disagree | Disagree | Neither agree nor disagree | Agree | Strongly Agree | Not Applicable | Prefer not to say |
| On a daily basis, I can finish a large number of tasks | Strongly disagree | Disagree | Neither agree nor disagree | Agree | Strongly Agree | Not Applicable | Prefer not to say |

In the last 6 months, during **work from home** **working hours**, how much of the time would you usually spend:

|  | Percentage (in each row, enter a number 0-100, total should add up to 100%) |
| --- | --- |
| Sitting? |  |
| Standing? |  |
| Walking? |  |
| Performing heavy labour or physically demanding tasks? |  |
| Prefer not to say (enter ‘100’ into textbox) |  |

On that same day during your **non-work time,** how much of the time would you usually spend:

|  | Percentage (in each row, enter a number 0-100, total should add up to 100%) |
| --- | --- |
| Sitting? |  |
| Standing? |  |
| Walking? |  |
| Performing heavy labour or physically demanding tasks? |  |
| Prefer not to say (enter ‘100’ into textbox) |  |

Is your organization planning on returning to the workplace within the next year?

- Yes, on a full-time basis
- Yes, but on a flexible (part-time) arrangement
- No, employees are staying remote
- Employees will get to choose their work location
- I do not know

Taking everything into account, how many **DAYS PER WEEK** would you prefer to work at home during a **NON-PANDEMIC TIME**?

- None
- 1
- 2
- 3
- 4
- Every day
- Prefer not to say

You indicated that you would prefer to work from home at least 1 or more days per week. What is the **primary reason** why you would prefer to continue working from home?

- I am more productive at home
- I am better able to balance my family/home and work commitments
- I enjoy the flexibility
- I am less stressed
- I save on commute time and money
- Other

*Please indicate the* other *reason you would like to continue working from home.*

You indicated that you would prefer not to work from home. What is the **primary reason** why you do not want to continue working from home?

- I am less productive at home
- I am less able to balance my family/home and work commitments
- I work longer hours
- Communication with my colleagues and supervisor regarding my work is lacking
- I do not have the appropriate workstation or technology to carry out my job requirements
- Other

*Please indicate the* other *reason you prefer to stop working from home.*

Taking everything into account, how many **DAYS PER WEEK** would you prefer to work at home when your **PERCEIVED RISK** of COVID-19 infection is **LOW**?

- None
- 1
- 2
- 3
- 4
- Every day
- Prefer not to say

Taking everything into account, how many **DAYS PER WEEK** would you prefer to work from home when your **PERCEIVED RISK** of COVID-19 infection is **HIGH**?

- None
- 1
- 2
- 3
- 4
- Every day
- Prefer not to say

You are now at the end of the survey. Thank you very much for your time.

- Would you like to be entered into a draw to win a $50 gift card? Yes No

You have indicated you would like to be entered into the draw. Please enter your email address. This personal information will only be used by the research team to send you the gift card. Once this has been done, your contact details will be removed from our records).

Email address:
