# Supplementary material for: Mental and physical health and well-being of canadian employees who were working from home during the COVID-19 pandemic
Source: BMC Public Health. 2022 Oct 31;22:1987. doi: 10.1186/s12889-022-14349-5 (PMC9619010; doi:10.1186/s12889-022-14349-5)
Supplement: Supplementary file 3 — Supplementary Material 3 [file 12889_2022_14349_MOESM3_ESM.docx]

**Contingency tables for McNemar-Bowker and McNemar’s tests**

**Supplementary Table 1.** Contingency table for McNemar-Bowker test to compare the workstation location between initial and follow-up surveys.

|  |  | **Follow-up** |  |  |  |
| --- | --- | --- | --- | --- | --- |
|  |  | Separate room | Separate room with interruptions | Work wherever | Total |
| **Initial** | Separate room | 225 | 19 | 8 | 252 |
|  | Separate room with interruptions | 18 | 42 | 5 | 65 |
|  | Work wherever | 23 | 7 | 35 | 65 |
|  | Total | 266 | 68 | 48 | 382 |

**Supplementary Table 2.** Contingency table for McNemar’s test to compare the usage of adjustable chairs between initial and follow-up surveys.

|  |  | **Follow-up** | |  |
| --- | --- | --- | --- | --- |
|  |  | No | Yes | Total |
| **Initial** | No | 69 | 44 | 113 |
|  | Yes | 23 | 245 | 268 |
|  | Total | 92 | 289 | 381 |

**Supplementary Table 3.** Contingency table for McNemar’s test to compare the usage of laptops between initial and follow-up surveys.

|  |  | **Follow-up** |  |  |
| --- | --- | --- | --- | --- |
|  |  | No | Yes | Total |
| **Initial** | No | 35 | 8 | 43 |
|  | Yes | 5 | 334 | 339 |
|  | Total | 40 | 342 | 382 |

**Supplementary Table 4.** Contingency table for McNemar-Bowker test to compare the usage of separate keyboard and/or mouse with laptops between initial and follow-up surveys.

|  |  | **Follow-up** |  |  |  |  |
| --- | --- | --- | --- | --- | --- | --- |
|  |  | Both keyboard and mouse | Keyboard only | Mouse only | Neither | Total |
| **Initial** | Both keyboard and mouse | 215 | 1 | 10 | 8 | 234 |
|  | Keyboard only | 0 | 1 | 1 | 0 | 2 |
|  | Mouse only | 15 | 0 | 41 | 7 | 63 |
|  | Neither | 18 | 2 | 11 | 46 | 77 |
|  | Total | 248 | 4 | 63 | 61 | 376 |

**Supplementary Table 5.** Contingency table for McNemar’s test to compare the usage of secondary monitors with laptops between initial and follow-up surveys.

|  |  | **Follow-up** | |  |
| --- | --- | --- | --- | --- |
|  |  | No | Yes | Total |
| **Initial** | No | 86 | 26 | 112 |
|  | Yes | 11 | 212 | 223 |
|  | Total | 97 | 238 | 335 |

**Supplementary Table 6.** Contingency table for McNemar’s test to compare the usage of desktops between initial and follow-up surveys.

|  |  | **Follow-up** |  |  |
| --- | --- | --- | --- | --- |
|  |  | No | Yes | Total |
| **Initial** | No | 203 | 32 | 235 |
|  | Yes | 40 | 107 | 147 |
|  | Total | 243 | 139 | 382 |

**Supplementary Table 7.** Contingency table for McNemar’s test to compare the usage of secondary screen with desktops between initial and follow-up surveys.

|  |  | **Follow-up** |  |  |
| --- | --- | --- | --- | --- |
|  |  | No | Yes | Total |
| **Initial** | No | 28 | 7 | 35 |
|  | Yes | 7 | 65 | 72 |
|  | Total | 35 | 72 | 107 |

**Supplementary Table 8.** Contingency table for McNemar’s test to compare the usage of phone/tablets between initial and follow-up surveys.

|  |  | **Follow-up** |  |  |
| --- | --- | --- | --- | --- |
|  |  | No | Yes | Total |
| **Initial** | No | 52 | 27 | 79 |
|  | Yes | 35 | 268 | 303 |
|  | Total | 87 | 295 | 382 |
