# Supplementary material for: Mental and physical health and well-being of canadian employees who were working from home during the COVID-19 pandemic
Source: BMC Public Health. 2022 Oct 31;22:1987. doi: 10.1186/s12889-022-14349-5 (PMC9619010; doi:10.1186/s12889-022-14349-5)
Supplement: Supplementary file 4 — Supplementary Material 4 [file 12889_2022_14349_MOESM4_ESM.docx]

**Frequency tables for questions asked only in the initial or follow-up survey**

**Supplementary Table 1.** Employment situation of participants based on questions asked only in initial survey.

|  | **Initial (n = 1617)** |
| --- | --- |
| **Frequency of WFH before COVID-19 ^a^** |  |
| None | 1242 (76.8%) |
| 1 day/week | 128 (7.9%) |
| 2 days/week | 84 (5.2%) |
| 3 days/week | 44 (2.7%) |
| 4 days/week | 28 (1.7%) |
| 5+ days/week | 90 (5.6%) |
| **Frequency of WFH during COVID-19** |  |
| 2 days/week | 54 (3.3%) |
| 3 days/week | 46 (2.8%) |
| 4 days/week | 111 (6.9%) |
| 5+ days/week | 1406 (87%) |
| **Average hours of WFH per week** |  |
| 14 hrs or less | 16 (1%) |
| 15-20 hrs | 21 (1.3%) |
| 21-25 hrs | 32 (2%) |
| 26-34 hrs | 79 (4.9%) |
| Full time | 1469 (90.8%) |
| **Number of months WFH before COVID-19 ^a, b^** | |
| 0-6 months | 1 (0.1%) |
| 6-12 months | 82 (5.1%) |
| More than 12 months | 40 (2.5%) |
| **Number of months WFH since COVID-19 ^c^** | 1605; 7.82 ± 2.14 |

^a^ The number of respondents who chose *Prefer not to say* as the response option are not presented; therefore, the sum of proportions is less than 100%.

^b^ Only respondents who worked from home for at least 1 day/week *before COVID-19* answered the question; therefore, the sum of proportions is less than 100%.

^c^ Participants who chose *Prefer not to say* as the response option were removed before calculating the mean and standard deviation.

**Supplementary Table 2.** Source of hardware and software technology as well as employees’ usage of software technology based on questions asked only in the initial survey.

|  | **Initial (n = 1617)** |
| --- | --- |
| **Employer-provided hardware technology** |  |
| Laptop | 1097 (67.8%) |
| Desktop | 344 (21.3%) |
| Separate keyboard | 730 (45.1%) |
| Mouse | 992 (61.3%) |
| Tablet | 463 (28.6%) |
| Other | 89 (5.5%) |
| **Employee-provided hardware technology** |  |
| Laptop | 267 (16.5%) |
| Desktop | 195 (12.1%) |
| Separate keyboard | 321 (19.9%) |
| Mouse | 475 (29.4%) |
| Tablet | 186 (11.5%) |
| Other | 100 (6.2%) |
| **Employer-provided software technology** |  |
| Online meeting platform | 1485 (91.8%) |
| Software program | 1346 (83.2%) |
| Access to organization network | 1445 (89.4%) |
| Other | 91 (5.6%) |
| **Employee-provided software technology** |  |
| Online meeting platform | 122 (7.5%) |
| Software program | 53 (3.3%) |
| Access to organization network | 92 (5.7%) |
| Other | 38 (2.4%) |
| **Software technology usage** |  |
| Video conferencing ^a^ |  |
| No | 137 (8.5%) |
| Yes | 1474 (91.2%) |
| Average hours spent on video conferencing or telephone ^a^ |  |
| Less than 1 hour | 585 (36.2%) |
| Between 1 and 2 hours | 503 (31.1%) |
| Between 2 and 3 hours | 261 (16.1%) |
| More than 3 hours | 253 (15.6%) |

^a^ The number of respondents who chose *Prefer not to say* as the response option are not presented; therefore, the sum of proportions is less than 100%.

**Supplementary Table 3.** Employment situation of participants based on questions asked only in follow-up survey.

|  | **Follow-up (n = 382)** |  |
| --- | --- | --- |
| **Became unemployed in the last 6 months** |  |  |
| No | 380 (99.5%) |  |
| Yes | 2 (0.5%) |  |
| **Started a new job in the last 6 months** |  |  |
| No | 361 (94.5%) |  |
| Yes | 21 (5.5%) |  |
| **Change in salary in the last 6 months ^a^** |  |  |
| Increased | 124 (32.5%) |  |
| Decreased | 11 (2.9%) |  |
| No changes | 219 (57.3%) |  |
| **Change in number of vacation/sick days used compared to pre-pandemic** |  |  |
| Decreased | 154 (40.3%) |  |
| Increased | 36 (9.4%) |  |
| No changes | 192 (50.3%) |  |
| **Satisfied by the amount of flexibility and sick days ^a^** |  |  |
| No | 51 (13.4%) |  |
| Yes | 325 (85.1%) |  |
| **Working arrangement in the last 6 months** |  |  |
| Return to workplace for a short time, then back to WFH | 11 (2.9%) |  |
| Split time between workplace and WFH | 47 (12.3%) |  |
| WFH the entire time | 307 (80.4%) |  |
| Other | 17 (4.5%) |  |

^a^ The number of respondents who chose *Prefer not to say* as the response option are not presented; therefore, the sum of proportions is less than 100%.

**Supplementary Table 4.** Participants’ primary reasons for WFH preferences and their organization’s return to work plan based on questions asked only in the follow-up survey.

|  | **Follow-up (n = 382)** |
| --- | --- |
| **Primary reason for preferring to continue to WFH ^a^** |  |
| Better able to balance work and family commitments | 67 (17.5%) |
| Enjoy the flexible work environment | 110 (28.8%) |
| Less stressed | 31 (8.1%) |
| More productive at home | 64 (16.8%) |
| Save on commute time and money | 71 (18.6%) |
| Other | 13 (3.4%) |
| **Primary reason for preferring not to continue to WFH ^b^** |  |
| Do not have appropriate workstation or technology for work | 3 (0.8%) |
| Lack communication with colleagues and supervisor regarding work | 3 (0.8%) |
| Less able to balance work and family commitments | 4 (1%) |
| Less productive at home | 5 (1.3%) |
| Work longer hours | 4 (1%) |
| Other | 5 (1.3%) |
| **Organization's return to work plan** |  |
| Do not know | 179 (46.9%) |
| Employees can choose work location | 15 (3.9%) |
| Staying remote | 23 (6%) |
| Returning to office but not full-time | 114 (29.8%) |
| Returning to office full-time | 51 (13.4%) |

^a^ Only participants who indicated that they would prefer to work from home in a *non-pandemic time* answered this question; therefore, the sum of proportions is less than 100%.

^b^ Only participants who indicated that they would prefer *not* to work from home in a *non-pandemic time* answered this question; therefore, the sum of proportions is less than 100%.
